# Investigating the Meat Pathway as a Source of Human Nontyphoidal *Salmonella* Bloodstream Infections and Diarrhea in East Africa

**DOI:** 10.1093/cid/ciaa1153

**Published:** 2020-08-10

**Authors:** John A Crump, Kate M Thomas, Jackie Benschop, Matthew A Knox, David A Wilkinson, Anne C Midwinter, Peninah Munyua, John B Ochieng, Godfrey M Bigogo, Jennifer R Verani, Marc-Alain Widdowson, Gerard Prinsen, Sarah Cleaveland, Esron D Karimuribo, Rudovick R Kazwala, Blandina T Mmbaga, Emanuel S Swai, Nigel P French, Ruth N Zadoks

**Affiliations:** 1 Centre for International Health, University of Otago, Dunedin, New Zealand; 2 Kilimanjaro Clinical Research Institute, Kilimanjaro Christian Medical Centre, Moshi, Tanzania; 3 Kilimanjaro Christian Medical University College, Moshi, Tanzania; 4 School of Veterinary Science, Massey University, Palmerston North, New Zealand; 5 Division of Global Health Protection, US Centers for Disease Control and Prevention, Nairobi, Kenya; 6 Centre for Global Health Research, Kenya Medical Research Institute, Kisumu, Kenya; 7 Institute of Tropical Medicine, Antwerp, Belgium; 8 School of People, Environment and Planning, Massey University, Palmerston North, New Zealand; 9 Institute of Biodiversity, Animal Health, and Comparative Medicine, College of Medical, Veterinary and Life Sciences, University of Glasgow, Glasgow, Scotland, United Kingdom; 10 College of Veterinary Medicine and Biomedical Sciences, Sokoine University of Agriculture, Morogoro, Tanzania; 11 Department of Veterinary Services, Ministry of Livestock and Fisheries, Dodoma, Tanzania; 12 Sydney School of Veterinary Science, University of Sydney, Sydney, Australia

**Keywords:** Eastern Africa, bacteremia, diarrhea, food, *Salmonella*

## Abstract

**Background:**

*Salmonella* Enteritidis and *Salmonella* Typhimurium are major causes of bloodstream infection and diarrheal disease in East Africa. Sources of human infection, including the role of the meat pathway, are poorly understood.

**Methods:**

We collected cattle, goat, and poultry meat pathway samples from December 2015 through August 2017 in Tanzania and isolated *Salmonella* using standard methods. Meat pathway isolates were compared with nontyphoidal serovars of *Salmonella enterica* (NTS) isolated from persons with bloodstream infections and diarrheal disease from 2007 through 2017 from Kenya by core genome multi-locus sequence typing (cgMLST). Isolates were characterized for antimicrobial resistance, virulence genes, and diversity.

**Results:**

We isolated NTS from 164 meat pathway samples. Of 172 human NTS isolates, 90 (52.3%) from stool and 82 (47.7%) from blood, 53 (30.8%) were *Salmonella* Enteritidis sequence type (ST) 11 and 62 (36.0%) were *Salmonella* Typhimurium ST313. We identified cgMLST clusters within *Salmonella* Enteritidis ST11, *Salmonella* Heidelberg ST15, *Salmonella* Typhimurium ST19, and *Salmonella* II 42:r:- ST1208 that included both human and meat pathway isolates. *Salmonella* Typhimurium ST313 was isolated exclusively from human samples. Human and poultry isolates bore more antimicrobial resistance and virulence genes and were less diverse than isolates from other sources.

**Conclusions:**

Our findings suggest that the meat pathway may be an important source of human infection with some clades of *Salmonella* Enteritidis ST11 in East Africa, but not of human infection by *Salmonella* Typhimurium ST313. Research is needed to systematically examine the contributions of other types of meat, animal products, produce, water, and the environment to nontyphoidal *Salmonella* disease in East Africa.

## Teaser

The meat pathway may be an important source of human invasive *Salmonella* Enteritidis ST11 infections in East Africa, but not of *Salmonella* Typhimurium ST313. Improvements to meat safety are warranted while research on sources for other nontyphoidal *Salmonella* infections continues.

Nontyphoidal serovars of *Salmonella enterica* (NTS) were associated with an estimated >153 million illnesses and >56 000 deaths worldwide in 2010 [1]. NTS are a leading cause of bloodstream infection in sub-Saharan Africa [2], occurring often in the absence of diarrhea and carrying a case fatality ratio of up to approximately 20% [3]. NTS bacteremia may be associated with human immunodeficiency virus (HIV) infection, recent or current malaria, and malnutrition [3]. Even after accounting for HIV-associated disease, the burden of NTS bacteremia in sub-Saharan Africa is substantial [4, 5]. The role of NTS in diarrheal disease in African countries is less clear. While approximately 9% of diarrheal illnesses and 11% of diarrheal deaths in the World Health Organization African region were attributed to NTS [1], NTS were isolated from <1% of stool samples from infants and young children with diarrhea at African sites in 1 large study of diarrheal disease [6]. Furthermore, it is common for NTS to be isolated no more often from the stool of infants and children with diarrhea than from community controls [6, 7]. Nonetheless, the acquisition of NTS in stool is likely to precede the development of invasive disease. While there has been relatively little work to characterize those NTS causing diarrheal disease in sub-Saharan Africa [8], *Salmonella* Typhimurium sequence type (ST) 313 [9] and several distinct clades of *Salmonella* Enteritidis ST11 [10, 11] strains predominate among bloodstream isolates from the region.

Whereas the typhoidal *Salmonella* serovars Typhi and Paratyphi A are human host–restricted, NTS are generally considered to have their reservoirs in nonhuman animals. Approximately half of global NTS infections are thought to be transmitted by food [1], with meat being a major food vehicle in high-income countries [12]. Unlike the situation in high-income countries, where foodborne disease surveillance is well developed and epidemiologic investigations inform control measures, there are few data on the major reservoirs, sources, and modes of transmission of NTS in Africa. The lack of epidemiologic data hampers control efforts. NTS may host generalists, such as *Salmonella* Typhimurium; exhibit degrees of host adaptation, such as the *Salmonella* Dublin adaptated to cattle; or host restriction, such as the *Salmonella* Gallinarum restricted to poultry [3]. A whole-genome sequencing analysis of *Salmonella* Typhimurium ST313 and *Salmonella* Enteritidis ST11 strains has demonstrated the inactivation of some genes [9, 11] that have been speculated to indicate adaptation towards a narrower ecologic niche, such as the human host [9]. However, other evidence, such as the ability of *Salmonella* Typhimurium ST313 to infect and cause disease in poultry [13], is a counterpoint to human host restriction. Furthermore, from a food safety perspective, NTS from both healthy animals and those with disease have the potential to enter the food chain directly on meat or through the contamination of produce and water. Several livestock species in Africa carry *Salmonella* Typhimurium and other NTS serovars, including cattle and poultry [14–16].

Developments in enteric pathogen epidemiology, including cluster-based inference and source attribution models that use microbial subtyping data to assign human infections to animal and environmental sources are being used to understand NTS epidemiologies in high-income countries [17]. These approaches have been proposed to investigate sources of NTS disease in Africa [18]. In order to understand the potential contribution of poultry and red meat to human NTS disease in sub-Saharan Africa, we studied NTS from livestock to retail meat along the meat pathway and from human bloodstream and enteric infections in Tanzania and Kenya, East Africa, where movement of livestock, food, and people is common. We used multi-locus sequence typing (MLST) cluster analysis to determine the genetic relatedness of isolates, in order to investigate the contribution of meat to human NTS infections.

## METHODS

### Study Setting and Sampling

**Figure 1.**
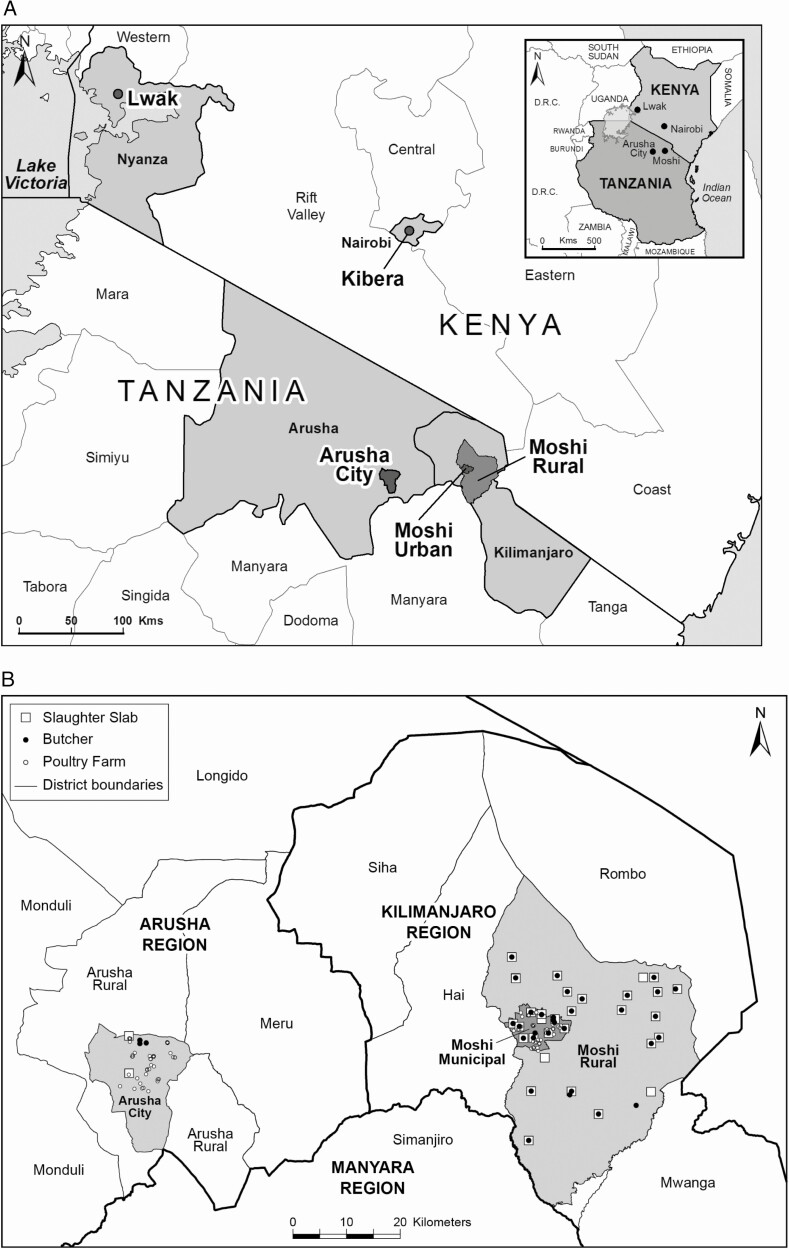
Map showing (*A*) data collection sites in Lwak and Kibera, Kenya, 2007–17, and (*B*) slaughter slab, butcher, and poultry farm locations sampled for nontyphoidal *Salmonella,* Arusha and Kilimanjaro Regions, northern Tanzania, 2015–17.

Meat pathway samples were collected from December 2015 through August 2017 in red meat slaughter and butcher facilities and on poultry farms in the Arusha Urban, Moshi Municipal, and Moshi Rural Districts of northern Tanzania (Figure 1). As described elsewhere, 10 live poultry on each of 80 poultry farms were sampled by cloacal swab and their farm environments were sampled by boot socks (Solar Biologicals Inc., Newark, NJ); 4 farms per ward were randomly selected from 10 wards each in the Arusha Urban and Moshi Municipal Districts [19]. Red meat slaughter facilities in the Arusha Urban and Moshi Municipal Districts were sampled between 2 and 25 times. Red meat slaughter and butcher facility environment swabs were taken from knives and cutting equipment, cutting boards, walls, sinks, hanging rails, and other solid surfaces with sterile cellulose sponge swabs that had been predosed with 10 mL buffered peptone water in stomacher bags (TSC Technical Service Consultants, Lancashire, UK). Boot socks were used to sample the facility floors. Liquid runoff from open waste drains was also collected, where available, in 60 mL sterile containers. Cloacal swabs were taken from poultry using Amies transport swabs (Sterilin Ltd, Newport, UK). At least 25 g of intestinal samples from cattle and goats were taken after slaughter. Carcasses of cattle and goats were swabbed at both the rump and the shoulder using both dry cotton-tipped swabs and cotton-tipped swabs moistened with Maximum Recovery Diluent (Oxoid), following the New Zealand Ministry for Primary Industries National Microbiological Database program protocol [20]. Metal carcass swab templates (100 cm^2^ for cattle and 25 cm^2^ for goats) were sterilized with 70% ethanol wipes and allowed to air dry between swabbings. Swab heads were snapped off into empty sterile 30 mL universal tubes (Greiner Bio-One Ltd, Gloucester, UK) for transport. Cattle and goat meat were obtained from meat sellers in the Arusha Urban, Moshi Municipal, and Moshi Rural Districts, whose meat was supplied by study slaughter facilities. Approximately 500 g of the lowest hanging section of cattle and goat meat on display was purchased and placed in a resealable plastic bag. All samples were transported in a cooler box with freezer packs to Kilimanjaro Clinical Research Institute Biotechnology Laboratory in Moshi for testing on the day of sampling.

Equal numbers of human bloodstream and diarrheal disease NTS isolates were sought from Kibera, Nairobi, and Lwak Mission Hospital, western Kenya. NTS were sequentially isolated from 2007 through 2017 from an ongoing population-based infectious disease surveillance system operated by the Kenya Medical Research Institute in collaboration with the US Centers for Disease Control and Prevention [21]. Isolates were identified as described previously [22], frozen and shipped to the Kilimanjaro Clinical Research Institute Biotechnology Laboratory in tryptone soya broth with 20% glycerol, and stored at −80°C.

### Isolation, Identification, Enumeration, and Antimicrobial Susceptibility Testing of *Salmonella* from the Meat Pathway

As described previously [19], isolation and identification of *Salmonella* was performed from meat pathway swabs, from 1 g intestinal samples and 25 g meat, to yield 1–5 presumptive *Salmonella* isolates identified per sample. Enumeration of *Salmonella* was performed on the day of sampling using a spiral plater (Wasp, Don Whitley, West Yorkshire, UK) for cloacal swabs, intestinal contents, meat samples, environmental samples, or a direct manual spread plate for carcass swabs. Xylose lysine deoxycholate agar (Oxoid) with 5 μg/mL novobiocin (Merck KGaA, Darmstadt, Germany) plates were inoculated, in duplicate, with 50 μl of freshly prepared homogenate. Plates were incubated overnight at 37 ± 2°C. Typical *Salmonella* colonies were counted manually and biochemically confirmed [19], and the number of *Salmonella* in the original sample was calculated. Routine phenotypic antimicrobial susceptibility testing was performed against amoxicillin/clavulanate, ampicillin, ceftazidime, ceftriaxone, chloramphenicol, ciprofloxacin, naladixic acid, and trimethoprim/sulfamethoxazole on environmental, poultry, and livestock isolates by disk diffusion, and interpreted to contemporary guidelines [23].

### Molecular Confirmation and DNA Preparation of *Salmonella* Isolates

*Salmonella* isolates from livestock, poultry, and their environments from northern Tanzania and from human blood and stool from Kenya were shipped to ^*m*^EpiLab, Hopkirk Research Institute, Massey University, Palmerston North, New Zealand. Following subculture, DNA was extracted from each isolate using the QiaAmp DNA minikit (Qiagen, Hilden, Germany). *Salmonella* isolates were confirmed by polymerase chain reaction targeting the *Salmonella* enterotoxin (*stn*) gene [24]. Libraries were prepared using an Illumina NexteraXT library preparation kit (Illumina, San Diego, CA) following the manufacturer’s instructions and were submitted to New Zealand Genomics Limited, University of Otago, Dunedin, New Zealand, for whole-genome sequencing using a HiSeq 2 × 125-bp PE v4 instrument (Illumina).

### Data Analysis

Illumina read data were cleaned using Trimmomatic version 0.38 [25]. Draft genomes were assembled using SPAdes version 3.11 [26]. Processed reads are publicly available on the National Center for Biotechnology Information Sequence Read Archive under BioProject ID PRJNA602741. Metadata are stored under BioSample accession numbers SAMN13905911–SAMN13906457 (Supplementary Table 1). Resistome and virulome profiles were assessed using ABRicate (https://github.com/tseemann/abricate) to query the ResFinder and the Virulence Factors (VFDB) databases [27, 28]. Annotation of antimicrobial resistance genes and resistance mechanisms was performed using the Resistance Gene Identifier algorithm web portal (RGI 5.0.0) and the comprehensive antibiotic resistance database (CARD 3.0.2) [29]. We employed criteria of “high quality/coverage” and “perfect and strict” hits only, and excluded “nudging of ≥ 95% identity loose hits to strict.” Assembly statistics were compiled using seqkit [30] as part of the Nullarbor pipeline [31]. We identified 7 gene STs using MLST (https://github.com/tseemann/mlst) [32]. Core genome MLST (cgMLST) types and serovar information were predicted using SISTR [33]. Allelic profiles were clustered using globally optimal eBURST (goeBURST) [34] in phylogenetic inference and data visualization for sequence based typing methods (PHYLOViZ) [35]. A distance threshold (T), expressed as the number of allelic differences for which isolates form the same cluster, was applied and used to generate goeBURST clusters at all possible similarity thresholds. The Neighbourhood Adjusted Wallace Coefficient (nAWC) [36], that examines the congruence of partitions between adjacent similarity thresholds (T), was used for cluster definition (https://github.com/theInnuendoProject/nAWC), to assess cluster grouping dynamics. We identified cgMLST clusters reflecting basic units in the overall *Salmonella* population structure, defined as the earliest point at which 5 or more consecutive thresholds yielded nAWC values > 0.99. To visualize the *Salmonella* population structure, we generated a minimum spanning tree using R packages “igraph” [37], “MLSTar” [38], “RColorBrewer [39],” “gplots [40],” and “ape” [41]. A tree displaying the relationship between ST type, resistome, and genotype was generated using the Interactive Tree of Life [42]. The circular dendrogram was generated by calculating a distance matrix based on the pairwise number of core genome allele differences between isolates, and clustering was done using Ward’s method [43]. ST diversity was estimated for each source by calculating the Simpson (1-D) and Shannon indices and plotting rarefaction curves. Both indices compare the diversity allowing for sample size, but the Shannon index places more emphasis on the richness, or number of different lineages, than the evenness, or how evenly distributed the different lineages are. The diversity indices with bootstrapped confidence intervals were calculated using the R package “vegetarian” version 1.2 [44], and the rarefaction curves were plotted using the R package “vegan” version 2.5–3 [45]. Human and meat pathway *Salmonella* Enteritidis strains were compared with previously described African and global lineages by cgMLST [11].

### Research Ethics

This study was approved by the Tanzania National Institutes for Medical Research National Research Ethics Coordinating Committee, the Kenya Medical Research Institute Scientific and Ethics Review Unit, the University of Otago Human Ethics Committee, and the University of Glasgow College of Medical, Veterinary, and Life Sciences Ethics Committee.

## RESULTS

### *Salmonella* from Poultry and Red Meat Pathways and from Humans

Of 164 meat pathway samples yielding *Salmonella*, 33 (20.1%) were from poultry farms, 32 (19.5%) from ruminant slaughter or butcher environments, 62 (37.8%) from cattle or their meat, and 37 (22.6%) from goats or their meat. Detailed information on specific sources and locations and enumeration among positive samples is shown in Table 1. The 164 meat pathway samples yielded 367 NTS isolates. Of 172 *Salmonella* isolates selected from humans, 90 (52.3%) were from the bloodstream and 82 (47.7%) were from the stool of patients with diarrhea.

**Table 1.** Numbers of samples positive for *Salmonella* and enumeration, northern Tanzania, 2015-17

	Arusha Urban			Moshi Municipal			Moshi Rural			*Salmonella* enumeration, log CFU/sample		Total		
	n/	N	(%)	n/	N	(%)	n/	N	(%)	Median	(range)	n/	N	(%)
Poultry farm environment	5/	40	(12.5)	9/	40	(22.5)	-	-	-	3.6	(3.0–5.1)	14/	80	(17.5)
Poultry cloaca	8/	393	(2.0)	11/	402	(2.7)	-	…	-	3.7	(2.3–4.2)	19/	795	(2.4)
Slaughter and butcher environment	15/	108	(13.9)	10/	71	(14.1)	7/	48	(14.6)	3.7	(3.0–5.1)	32/	227	(14.1)
Cattle intestinal	1/	114	(.9)	2/	93	(2.2)	0/	128	(0)	2.3	(2.3–2.3)	3/	335	(.9)
Goat intestinal	0/	139	(0)	6/	84	(7.1)	1/	10	(10.0)	2.9	(2.6–3.3)	7/	233	(3.0)
Cattle carcass	4/	105	(3.8)	0/	50	(0)	1/	119	(.8)	0^a^	(0–0)	5/	274	(1.8)
Goat carcass	0/	134	(0)	6/	40	(15.0)	0/	12	(0)	0^a^	(0–0)	6/	186	(3.2)
Cattle meat	19/	180	(10.5)	21/	140	(15.0)	14/	143	(9.8)	3.4	(3.4–5.4)	54/	463	(11.7)
Goat meat	13/	118	(11.0)	10/	76	(13.2)	1/	11	(9.1)	3.9	(3.1–4.3)	24/	205	(11.7)
TOTAL	65/	1331	(4.9)	75/	996	(7.5)	24/	471	(5.1)	-	-	164/	2798	(5.8)

Data are from poultry cloaca, poultry farm environment, cattle and goat intestinal, carcass, meat, and slaughter and butcher environment samples in the Arusha Urban, Moshi Municipal, and Moshi Rural Districts, 2015–17.

Abbreviations: **-**, not applicable; CFU, colony forming units.

^a^Below level of enumeration in all samples.

### *Salmonella* sequence types, serovars, and diversity

Of 539 NTS isolates, 91 (16.9%) were *Salmonella* Typhimurium and 78 (14.5%) were *Salmonella* Enteritidis (Supplementary Table 2). Of 72 allelic profiles identified among all isolates, 17 (23.6%) were of previously undescribed STs. The predominant STs were *Salmonella* Enteritidis ST11 (n = 78) and *Salmonella* Typhimurium ST313 (n = 62; Table 2; Supplementary Table 2). Of the 8 sample types, *Salmonella* Enteritidis ST11 was found in 7, being absent only from the slaughter and butcher environment, whereas *Salmonella* Typhimurium ST313 was found only from human stool and blood. *Salmonella* Orion ST639 was found in 4 exclusively nonhuman sources.

**Table 2.** *Salmonella* sequence types and serovars, East Africa, 2007-17

		Sample type																
		Poultry farm environment		Poultry cloaca		Slaughter and butcher environment		Cattle and goat intestinal samples		Cattle and goat carcass		Cattle and goat meat		Human feces		Human blood		
*Salmonella* sequence type	*Salmonella* serovar	n	(%)	n	(%)	n	(%)	n	(%)	n	(%)	n	(%)	n	(%)	n	(%)	Total
11	Enteritidis	6	(26.1)	9	(27.3)	0	(0)	4	(28.6)	2	(18.2)	4	(4.9)	21	(35.6)	32	(39.5)	78
313	Typhimurium	0	(0)	0	(0)	0	(0)	0	(0)	0	(0)	0	(0)	17	(28.8)	45	(55.6)	62
639	Orion	0	(0)	0	(0)	7	(14.6)	2	(14.3)	2	(18.2)	22	(26.8)	0	(0)	0	(0)	33
1208	II 42:r:-	0	(0)	0	(0)	5	(10.4)	0	(0)	4	(36.4)	18	(22)	1	(1.7)	0	(0)	28
19	Typhimurium	1	(4.3)	4	(12.1)	1	(2.1)	3	(21.4)	1	(9.1)	3	(3.7)	9	(15.3)	2	(2.5)	24
27	Saintpaul	2	(8.7)	0	(0)	2	(4.2)	0	(0)	2	(18.2)	11	(13.4)	1	(1.7)	0	(0)	18
16	Virchow	3	(13)	3	(9.1)	0	(0)	0	(0)	0	(0)	7	(8.5)	1	(1.7)	1	(1.2)	15
166	Newport	5	(21.7)	6	(18.2)	1	(2.1)	0	(0)	0	(0)	2	(2.4)	0	(0)	0	(0)	14
22	Braenderup	0	(0)	0	(0)	14	(29.2)	0	(0)	0	(0)	0	(0)	0	(0)	0	(0)	14
15	Heidelberg	0	(0)	0	(0)	0	(0)	0	(0)	0	(0)	4	(4.9)	9	(15.3)	1	(1.2)	14
912	Karamoja	0	(0)	0	(0)	7	(14.6)	2	(14.3)	0	(0)	5	(6.1)	0	(0)	0	(0)	14
198	Kentucky	2	(8.7)	7	(21.2)	0	(0)	0	(0)	0	(0)	4	(4.9)	0	(0)	0	(0)	13
2533	Durban	4	(17.4)	4	(12.1)	0	(0)	3	(21.4)	0	(0)	2	(2.4)	0	(0)	0	(0)	13
Unknown	II 1,4,12,27:e,n,x:e,n,x	0	(0)	0	(0)	11	(22.9)	0	(0)	0	(0)	0	(0)	0	(0)	0	(0)	11
TOTAL		23	(100.0)	33	(100.0)	48	(100.0)	14	(100.0)	11	(100.0)	82	(100.0)	59	(100.0)	81	(100.0)	351

Data are by sample source for types with at least 15 isolates, East Africa, 2007–17. Details of other sequence types and serovars are available in Supplementary Table 2.

A comparison of the diversity of 7-gene MLSTs between different sample types, using rarefaction curves and the Simpson and Shannon indices, showed the highest diversity was associated with isolates from cattle and goat meat, followed by isolates from the red meat slaughter and butcher environment (Supplementary Table 3; Supplementary Figure 1). In contrast, the lowest diversity was associated with isolates from human blood. Blood isolates were significantly less diverse than the population isolated from human stool (Supplementary Table 3).

### Core Genome Multi-Locus Sequence Types

**Figure 2.**
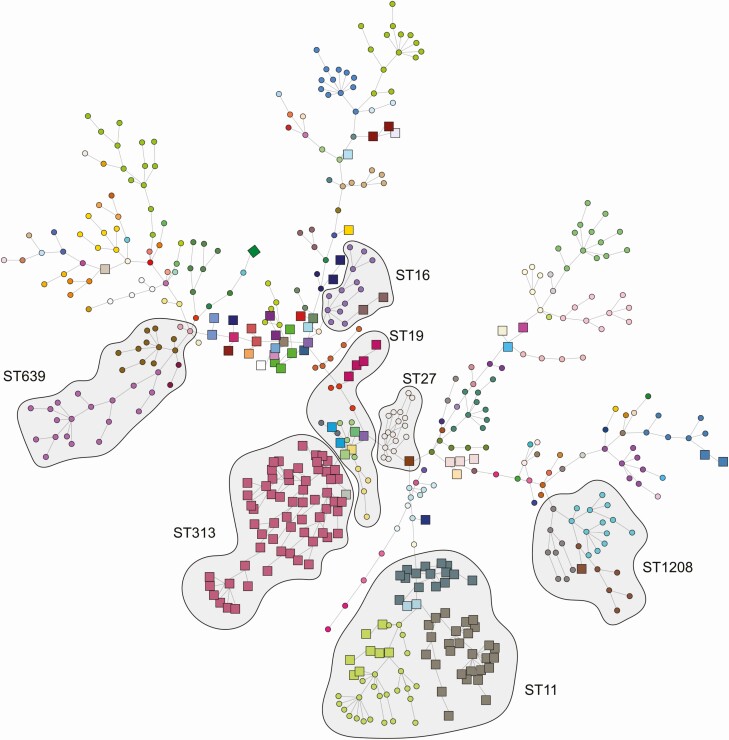
Minimum spanning tree based on *Salmonella* core genome multi-locus sequence type profiles overlaid with common 7-gene multi-locus sequence type clusters, East Africa, 2007–17. Human isolates are presented as squares and isolates from nonhuman sources are presented as circles. Shading represents 7-gene multi-locus sequence type clusters. Node colors differentiate cgMLST groupings, determined using globally optimal electronic based upon related sequence types (goeBURST) [34] in phylogenetic inference and data visualization for sequence based typing methods (PHYLOViZ) [35]. Abbreviations: cgMLST, core genome multi-locus sequence typing; ST, sequence type; ST11: *Salmonella* Enteritidis; ST313 and ST19: *Salmonella* Typhimurium; ST639: *Salmonella* Orion; ST1208: *Salmonella* II 42:r:-; ST27: *Salmonella* Saintpaul; ST16: *Salmonella* Virchow.

The cgMLST analyses using nAWC resulted in a cutoff of 38 allelic differences and 157 separate clusters, ranging in size from 1 to 65 isolates. The *Salmonella* population structure and cgMLST sequence types are shown in Figure 2. Similar to 7-gene MLST findings, sources of cgMLST clusters exhibited contrasting cluster-related patterns. Some cgMLSTs were found in all sample types, while others were restricted to human samples, or nonhuman samples. Within *Salmonella* STs 11, 16, 19, 27, and 1208, we identified cgMLST clusters that included both human and meat pathway isolates. When compared with previously described clades [11], *Salmonella* Enteritidis ST11 cgMLST clusters of similar human and meat pathway isolates belonged to the so-called global epidemic clade, rather than to Africa-restricted clades.

### Antimicrobial Resistance and Resistome

Antimicrobial susceptibility results and resistome profiles for 49 resistance genes from 9 antimicrobial classes are shown in Supplementary Table 4. Phenotypic antimicrobial susceptibility results of human isolates are reported elsewhere [46]. Of 539 isolates, resistance genes to aminoglycosides were found in 133 (24.7%), beta-lactams in 105 (19.5%), chloramphenicol in 136 (25.2%), trimethoprim in 97 (18.0%), sulphonamides in 131 (24.3%), and tetracycline in 102 (18.9%). Resistance genes to fosfomycin, macrolides, and quinolones were present in ≤11 isolates. Human blood isolates had the widest range of resistance genes, with 69 (84.1%) of 82 isolates having genes for 5 or more resistance classes (Supplementary Figure 2). *Salmonella* bearing genes for resistance to 2 or more antimicrobial classes were found in 14 (35.9%) of 39 poultry cloacae, 13 (38.2%) of 34 poultry farm environment, 29 (32.2%) of 90 human stool samples, and <10% of other sample sources (Supplementary Figure 2; Supplementary Figure 3; Supplementary Figure 4). The median numbers of resistance gene classes found in isolates from human, poultry, and ruminant samples were 5 (interquartile range [IQR], 0–6), 0 (IQR, 0–3), and 0 (IQR, 0–0), respectively.

### Virulome

Virulome profiles of the 539 isolates tested identified 153 virulence genes in total, of which 26 (17.0%) were considered major [28], including those associated with adherence (*sinH*, *ratB*, *pef*, *ipf*, genes associated withType 1 fimbriae, *shdA*, and *misL*), magnesium uptake (*mgtBC*), resistance to an antimicrobial peptide produced by macrophages (*mig-14*) [47], serum resistance (*rck*), an oxidative stress defense protein (*sodCI*), and other proteins (*spv* and *cdtB*). The median (IQR) of major virulence genes found in isolates derived from human, poultry, and ruminant samples were 26 (19–26), 18 (17–18) and 13 (12–17), respectively (Supplementary Figure 5).

## DISCUSSION

We demonstrated that NTS from East Africa, while relatively uncommon in cattle, goat, and poultry intestinal samples, were highly prevalent in the slaughter and butcher environment and in cattle and goat meat. *Salmonella* Enteritidis ST11 and *Salmonella* Typhimurium ST313 predominated among isolates from persons with bloodstream infection and diarrhea. By cgMLST, *Salmonella* Enteritidis ST11 from humans, belonging to the so-called global epidemic clade [11], and other less common *Salmonella* serovars and sequence types were clustered closely with strains isolated from the cattle, goat, and poultry meat pathway. However, *Salmonella* Typhimurium ST313 was not isolated from any meat pathway sample. Taken together, our findings suggest that the meat pathway may be an important source of human *Salmonella* Enteritidis ST11 infection in East Africa, but not of human *Salmonella* Typhimurium ST313 infection.

As in other regions, we confirm that in East Africa *Salmonella enterica* serovars Enteritidis and Typhimurium are leading causes of NTS invasive and diarrheal disease in humans [1]. Consistent with studies from high-income countries [48, 49], our cgMLST data suggest that both poultry and red meat may be sources of human *Salmonella* Enteritidis ST11 infections in East Africa. Notably, *Salmonella* Enteritidis ST11 that were highly similar between meat pathway and human disease isolates belonging to the so-called global epidemic clade rather than to Africa-restricted clades [11], leaving open questions about sources of Africa-restricted *Salmonella* Enteritidis ST11 clades. While we found *Salmonella* Typhimurium in all components of the meat pathway tested, no *Salmonella* Typhimurium ST313 were isolated from the meat pathway. While relatively few studies from African countries have examined *Salmonella* Typhimurium sequence types in nonhuman sources [50, 51], the existence of a nonhuman reservoir for *Salmonella* Typhimurium ST313, if any, remains to be established.

NTS was isolated throughout the meat pathway in northern Tanzania, including on a small proportion of carcasses and >10% of retail cattle and goat meat. While the prevalence of NTS was low in livestock intestinal samples and poultry cloacal samples, it was higher in poultry, livestock slaughter, and butcher environments. These findings point to the importance of cooking meat well prior to consumption, and suggest that the contamination of meat during slaughter and butchering from a range of sources may contribute substantially to contamination by *Salmonella* of retail meat in this setting.

Our study has a number of limitations. First, our meat pathway research and human disease surveillance were not co-located, and human disease isolates were collected over a period that extended beyond the period of meat pathway data collection. While movement of livestock, food, and people is common in East Africa, location and time differences may have reduced our ability to attribute human infections to meat pathway sources. Second, our research was limited to cattle and goat meat pathways, and the poultry component was restricted to live chickens and their environments. Although cattle, goats, and poultry are the major sources of meat in Tanzania, we cannot exclude roles for pigs, sheep, and other species in the epidemiology of human NTS infections. Finally, rarefaction curves did not plateau, suggesting that more diversity likely exists that was not sampled by our study.

In conclusion, we demonstrate that nontyphoidal *Salmonella* is common in the meat pathway in Tanzania, especially in slaughter and butcher environments, and as a contaminant of retail meat. MLST cluster analyses suggest that the meat pathway likely contributes to both human bloodstream infections and diarrheal disease due to *Salmonella* Enteritidis ST11 and to other less common *Salmonella* serovars and sequence types, including *Salmonella* Typhimurium other than ST313. However, we did not find evidence of a contribution to *Salmonella* Typhimurium ST313 infections. In addition to studies in humans, more research is needed to systematically examine the contribution of other types of meat, animal products, produce, water, and environmental exposures as potential sources of NTS disease in East Africa.

## Supplementary Data

Supplementary materials are available at *Clinical Infectious Diseases* online. Consisting of data provided by the authors to benefit the reader, the posted materials are not copyedited and are the sole responsibility of the authors, so questions or comments should be addressed to the corresponding author.

ciaa1153_suppl_Supplementary_Figure_1

ciaa1153_suppl_Supplementary_Figure_2

ciaa1153_suppl_Supplementary_Figure_3

ciaa1153_suppl_Supplementary_Figure_4

ciaa1153_suppl_Supplementary_Table_S1

ciaa1153_suppl_Supplementary_Table_S2

ciaa1153_suppl_Supplementary_Table_S3

ciaa1153_suppl_Supplementary_Table_S4

## Notes

***Acknowledgments.*** The authors thank members of the Tanzania field team, including Matayo L. Melubo, Rigobert A. Tarimo, and Fadhili I. Mshana; members of the laboratory team, including Ephrasia A. Hugho, Nelson B. Amani, Victor V. Mosha, Elizabeth A. Kasagama; postgraduate students Emmanuel Sindiyo and Ruth Maganga; the Kenya Medical Research Institute Center for Global Health Research for providing human stool and blood *Salmonella* isolates; Dr Michael C. Shand, PhD, University of Glasgow, Glasgow, United Kingdom, for mapping; Dr Linda Waldman, PhD, and Ms Tabitha Hrynick, MA, Institute for Development Studies, University of Sussex, Brighton, United Kingdom, and Dr Joanne P. Sharp, PhD, University of Saint Andrews, Scotland, for critical qualitative research that informed the design of the study; Dr Gary C. Barker, PhD, for related work on a model framework for the spread of nontyphoidal *Salmonella* in the meat pathway in northern Tanzania; Dr Margaret A. Davis, DVM, MPH, PhD, Washington State University, Pullman, WA, and Dr Niwael J. Mtui-Malamsha, BVM, MVM, PhD, Ministry of Livestock and Fisheries Development, Dodoma, Tanzania, for related meat safety policy review.

***Financial support.*** This work was supported by the UK Biotechnology and Biological Sciences Research Council, the UK Department for International Development, the UK Economic and Social Research Council, the UK Medical Research Council, the UK Natural Environment Research Council, and the UK Defense Science and Technology Laboratory through the Zoonoses and Emerging Livestock Systems programme (grant numbers BB/L017679/1 and BB/N503563/1).

***Potential Conflicts of Interest.*** D. A. W. received support from the New Zealand Food Safety Science and Research Centre. N. P. F. receives financial support as Director of New Zealand Food Safety Science and Research Centre, outside the submitted work. All other authors report no potential conflicts. All authors have submitted the ICMJE Form for Disclosure of Potential Conflicts of Interest. Conflicts that the editors consider relevant to the content of the manuscript have been disclosed.

Presented in part: 10th International Conference of Typhoid and Other Invasive Salmonelloses, Kampala, Uganda, 4–6 April 2017.
