# Supplementary material for: Investigating the Meat Pathway as a Source of Human Nontyphoidal Salmonella Bloodstream Infections and Diarrhea in East Africa
Source: Clin Infect Dis. 2020 Aug 10;73(7):e1570–8. doi: 10.1093/cid/ciaa1153 (PMC8492120; doi:10.1093/cid/ciaa1153)
Supplement: ciaa1153_suppl_Supplementary_Figure_1 [file ciaa1153_suppl_supplementary_figure_1.docx]

**Supplementary Figure 1. Rarefaction graph of 7-gene multi-locus sequence types by sample type, East Africa, 2007-17**
