# Supplementary material for: Investigating the Meat Pathway as a Source of Human Nontyphoidal Salmonella Bloodstream Infections and Diarrhea in East Africa
Source: Clin Infect Dis. 2020 Aug 10;73(7):e1570–8. doi: 10.1093/cid/ciaa1153 (PMC8492120; doi:10.1093/cid/ciaa1153)
Supplement: ciaa1153_suppl_Supplementary_Figure_2 [file ciaa1153_suppl_supplementary_figure_2.docx]

**Supplementary Figure 2. Proportion of *Salmonella* isolates by sample type with resistance genes to antimicrobial classes, East Africa, 2007-17**

Number of isolates for sample type is in parentheses.
