# Supplementary material for: Investigating the Meat Pathway as a Source of Human Nontyphoidal Salmonella Bloodstream Infections and Diarrhea in East Africa
Source: Clin Infect Dis. 2020 Aug 10;73(7):e1570–8. doi: 10.1093/cid/ciaa1153 (PMC8492120; doi:10.1093/cid/ciaa1153)
Supplement: ciaa1153_suppl_Supplementary_Figure_3 [file ciaa1153_suppl_supplementary_figure_3.docx]

**Supplementary Figure 3. Presence and absence of resistance genes to aminoglycoside (A), beta-lactam (B), phenicol (C), sulphonamide (D), tetracycline (E), trimethoprim (F), in *Salmonella* isolates by sample type, East Africa, 2007-17**

**A**

**B**

**C**

**D**

**E**

**F**

Number of isolates for sample type is in parentheses
