# Supplementary material for: Investigating the Meat Pathway as a Source of Human Nontyphoidal Salmonella Bloodstream Infections and Diarrhea in East Africa
Source: Clin Infect Dis. 2020 Aug 10;73(7):e1570–8. doi: 10.1093/cid/ciaa1153 (PMC8492120; doi:10.1093/cid/ciaa1153)
Supplement: ciaa1153_suppl_Supplementary_Table_S1 [file ciaa1153_suppl_supplementary_table_s1.docx]

**Supplementary Table 1. List of processed *Salmonella enterica* whole genome sequence reads available on the National Center for Biotechnology Information Sequence Read Archive under BioProject ID PRJNA602741, East Africa, 2007-17**

| **Isolate** | **BioProject** | **BioSample** | **Accession** | **Organism** |
| --- | --- | --- | --- | --- |
| HAZEL-001 | PRJNA602741 | SAMN13905911 | JAAHSW000000000 | *Salmonella enterica* subsp. *enterica* serovar Enteritidis HAZEL-001 |
| HAZEL-002 | PRJNA602741 | SAMN13905912 | JAAHSV000000000 | *Salmonella enterica* subsp. *enterica* serovar Enteritidis HAZEL-002 |
| HAZEL-003 | PRJNA602741 | SAMN13905913 | JAAHSU000000000 | *Salmonella enterica* subsp. *enterica* serovar Typhimurium HAZEL-003 |
| HAZEL-004 | PRJNA602741 | SAMN13905914 | JAAHST000000000 | *Salmonella enterica* subsp. *enterica* serovar Typhimurium HAZEL-004 |
| HAZEL-005 | PRJNA602741 | SAMN13905915 | JAAHSS000000000 | *Salmonella enterica* subsp. *enterica* serovar Karamoja HAZEL-005 |
| HAZEL-006 | PRJNA602741 | SAMN13905916 | JAAHSR000000000 | *Salmonella enterica* subsp. *enterica* serovar Karamoja HAZEL-006 |
| HAZEL-007 | PRJNA602741 | SAMN13905917 | JAAHSQ000000000 | *Salmonella enterica* subsp. *enterica* serovar Orion HAZEL-007 |
| HAZEL-008 | PRJNA602741 | SAMN13905918 | JAAHSP000000000 | *Salmonella enterica* subsp. *enterica* serovar Orion HAZEL-008 |
| HAZEL-009 | PRJNA602741 | SAMN13905919 | JAAHSO000000000 | *Salmonella enterica* subsp. *enterica* serovar Durban HAZEL-009 |
| HAZEL-010 | PRJNA602741 | SAMN13905920 | JAAHSN000000000 | *Salmonella enterica* subsp. *enterica* serovar Durban HAZEL-010 |
| HAZEL-011 | PRJNA602741 | SAMN13905921 | JAAHSM000000000 | *Salmonella enterica* subsp. *enterica* serovar Hadar HAZEL-011 |
| HAZEL-012 | PRJNA602741 | SAMN13905922 | JAAHSL000000000 | *Salmonella enterica* subsp. *enterica* serovar Hadar HAZEL-012 |
| HAZEL-013 | PRJNA602741 | SAMN13905923 | JAAHSK000000000 | *Salmonella enterica* subsp. *enterica* serovar Enteritidis HAZEL-013 |
| HAZEL-014 | PRJNA602741 | SAMN13905924 | JAAHSJ000000000 | *Salmonella enterica* subsp. *enterica* serovar Enteritidis HAZEL-014 |
| HAZEL-015 | PRJNA602741 | SAMN13905925 | JAAHSI000000000 | *Salmonella enterica* subsp. *enterica* serovar Chandans HAZEL-015 |
| HAZEL-016 | PRJNA602741 | SAMN13905926 | JAAHSH000000000 | *Salmonella enterica* subsp. *enterica* serovar Johannesburg HAZEL-016 |
| HAZEL-017 | PRJNA602741 | SAMN13905927 | JAAHSG000000000 | *Salmonella enterica* subsp. *enterica* serovar Johannesburg HAZEL-017 |
| HAZEL-018 | PRJNA602741 | SAMN13905928 | JAAHSF000000000 | *Salmonella enterica* subsp. *enterica* serovar Kiambu HAZEL-018 |
| HAZEL-020 | PRJNA602741 | SAMN13905930 | JAAHSD000000000 | *Salmonella enterica* subsp. *enterica* serovar Kiambu HAZEL-020 |
| HAZEL-021 | PRJNA602741 | SAMN13905931 | JAAHSC000000000 | *Salmonella enterica* subsp. *enterica* serovar Kiambu HAZEL-021 |
| HAZEL-022 | PRJNA602741 | SAMN13905932 | JAAHSB000000000 | *Salmonella enterica* subsp. *enterica* serovar Bahrenfeld HAZEL-022 |
| HAZEL-023 | PRJNA602741 | SAMN13905933 | JAAHSA000000000 | *Salmonella enterica* subsp. *enterica* serovar Bahrenfeld HAZEL-023 |
| HAZEL-024 | PRJNA602741 | SAMN13905934 | JAAHRZ000000000 | *Salmonella enterica* subsp. *salamae* serovar 42:r:- HAZEL-024 |
| HAZEL-025 | PRJNA602741 | SAMN13905935 | JAAHRY000000000 | *Salmonella enterica* subsp. *enterica* serovar Leoben HAZEL-025 |
| HAZEL-026 | PRJNA602741 | SAMN13905936 | JAAHRX000000000 | *Salmonella enterica* subsp. *salamae* serovar 42:r:- HAZEL-026 |
| HAZEL-027 | PRJNA602741 | SAMN13905937 | JAAHRW000000000 | *Salmonella enterica* subsp. *salamae* serovar 42:r:- HAZEL-027 |
| HAZEL-028 | PRJNA602741 | SAMN13905938 | JAAHRV000000000 | *Salmonella enterica* subsp. *salamae* serovar 42:r:- HAZEL-028 |
| HAZEL-029 | PRJNA602741 | SAMN13905939 | JAAHRU000000000 | *Salmonella enterica* subsp. *salamae* serovar 42:r:- HAZEL-029 |
| HAZEL-030 | PRJNA602741 | SAMN13905940 | JAAHRT000000000 | *Salmonella enterica* subsp. *enterica* serovar Livingstone HAZEL-030 |
| HAZEL-031 | PRJNA602741 | SAMN13905941 | JAAHRS000000000 | *Salmonella enterica* subsp. *enterica* serovar Saintpaul HAZEL-031 |
| HAZEL-032 | PRJNA602741 | SAMN13905942 | JAAHRR000000000 | *Salmonella enterica* subsp. *enterica* serovar Indiana HAZEL-032 |
| HAZEL-033 | PRJNA602741 | SAMN13905943 | JAAHRQ000000000 | *Salmonella enterica* subsp. *enterica* serovar Indiana HAZEL-033 |
| HAZEL-034 | PRJNA602741 | SAMN13905944 | JAAHRP000000000 | *Salmonella enterica* subsp. *enterica* HAZEL-034 |
| HAZEL-035 | PRJNA602741 | SAMN13905945 | JAAHRO000000000 | *Salmonella enterica* subsp. *enterica* HAZEL-035 |
| HAZEL-036 | PRJNA602741 | SAMN13905946 | JAAHRN000000000 | *Salmonella enterica* subsp. *enterica* serovar Orion HAZEL-036 |
| HAZEL-037 | PRJNA602741 | SAMN13905947 | JAAHRM000000000 | *Salmonella enterica* subsp. *enterica* serovar Orion HAZEL-037 |
| HAZEL-038 | PRJNA602741 | SAMN13905948 | JAAHRL000000000 | *Salmonella enterica* subsp. *enterica* serovar Newport HAZEL-038 |
| HAZEL-039 | PRJNA602741 | SAMN13905949 | JAAHRK000000000 | *Salmonella enterica* subsp. *enterica* serovar Newport HAZEL-039 |
| HAZEL-040 | PRJNA602741 | SAMN13905950 | JAAHRJ000000000 | *Salmonella enterica* subsp. *salamae* serovar 42:r:- HAZEL-040 |
| HAZEL-041 | PRJNA602741 | SAMN13905951 | JAAHRI000000000 | *Salmonella enterica* subsp. *salamae* serovar 42:r:- HAZEL-041 |
| HAZEL-042 | PRJNA602741 | SAMN13905952 | JAAHRH000000000 | *Salmonella enterica* subsp. *enterica* serovar Kentucky HAZEL-042 |
| HAZEL-043 | PRJNA602741 | SAMN13905953 | JAAHRG000000000 | *Salmonella enterica* subsp. *enterica* serovar Kentucky HAZEL-043 |
| HAZEL-044 | PRJNA602741 | SAMN13905954 | JAAHRF000000000 | *Salmonella enterica* subsp. *enterica* serovar Orion HAZEL-044 |
| HAZEL-045 | PRJNA602741 | SAMN13905955 | JAAHRE000000000 | *Salmonella enterica* subsp. *enterica* serovar Orion HAZEL-045 |
| HAZEL-046 | PRJNA602741 | SAMN13905956 | JAAHRD000000000 | *Salmonella enterica* subsp. *enterica* serovar Poona HAZEL-046 |
| HAZEL-047 | PRJNA602741 | SAMN13905957 | JAAHRC000000000 | *Salmonella enterica* subsp. *enterica* serovar Poona HAZEL-047 |
| HAZEL-048 | PRJNA602741 | SAMN13905958 | JAAHRB000000000 | *Salmonella enterica* subsp. *salamae* serovar 42:r:- HAZEL-048 |
| HAZEL-049 | PRJNA602741 | SAMN13905959 | JAAHRA000000000 | *Salmonella enterica* subsp. *salamae* serovar 42:r:- HAZEL-049 |
| HAZEL-050 | PRJNA602741 | SAMN13905960 | JAAHQZ000000000 | *Salmonella enterica* subsp. *enterica* serovar Kentucky HAZEL-050 |
| HAZEL-051 | PRJNA602741 | SAMN13905961 | JAAHQY000000000 | *Salmonella enterica* subsp. *enterica* serovar Kentucky HAZEL-051 |
| HAZEL-052 | PRJNA602741 | SAMN13905962 | JAAHQX000000000 | *Salmonella enterica* subsp. *enterica* serovar Virchow HAZEL-052 |
| HAZEL-053 | PRJNA602741 | SAMN13905963 | JAAHQW000000000 | *Salmonella enterica* subsp. *enterica* serovar Virchow HAZEL-053 |
| HAZEL-054 | PRJNA602741 | SAMN13905964 | JAAHQV000000000 | *Salmonella enterica* subsp. *enterica* serovar Virchow HAZEL-054 |
| HAZEL-055 | PRJNA602741 | SAMN13905965 | JAAHQU000000000 | *Salmonella enterica* subsp. *enterica* serovar Virchow HAZEL-055 |
| HAZEL-056 | PRJNA602741 | SAMN13905966 | JAAHTA000000000 | *Salmonella enterica* subsp. *enterica* serovar Virchow HAZEL-056 |
| HAZEL-057 | PRJNA602741 | SAMN13905967 | JAAHQT000000000 | *Salmonella enterica* subsp. *enterica* serovar Virchow HAZEL-057 |
| HAZEL-058 | PRJNA602741 | SAMN13905968 | JAAHQS000000000 | *Salmonella enterica* subsp. *enterica* serovar Virchow HAZEL-058 |
| HAZEL-059 | PRJNA602741 | SAMN13905969 | JAAHQR000000000 | *Salmonella enterica* subsp. *enterica* serovar Poona HAZEL-059 |
| HAZEL-060 | PRJNA602741 | SAMN13905970 | JAAHQQ000000000 | *Salmonella enterica* subsp. *salamae* serovar 42:r:- HAZEL-060 |
| HAZEL-061 | PRJNA602741 | SAMN13905971 | JAAHQP000000000 | *Salmonella enterica* subsp. *salamae* serovar 42:r:- HAZEL-061 |
| HAZEL-062 | PRJNA602741 | SAMN13905972 | JAAHQO000000000 | *Salmonella enterica* subsp. *enterica* serovar Kentucky HAZEL-062 |
| HAZEL-063 | PRJNA602741 | SAMN13905973 | JAAHQN000000000 | *Salmonella enterica* subsp. *enterica* serovar Kentucky HAZEL-063 |
| HAZEL-064 | PRJNA602741 | SAMN13905974 | JAAHQM000000000 | *Salmonella enterica* subsp. *enterica* serovar Enteritidis HAZEL-064 |
| HAZEL-065 | PRJNA602741 | SAMN13905975 | JAAHQL000000000 | *Salmonella enterica* subsp. *enterica* serovar Enteritidis HAZEL-065 |
| HAZEL-066 | PRJNA602741 | SAMN13905976 | JAAHUH000000000 | *Salmonella enterica* subsp. *salamae* serovar 1,13,23:z:1,5 HAZEL-066 |
| HAZEL-067 | PRJNA602741 | SAMN13905977 | JAAHUG000000000 | *Salmonella enterica* subsp. *salamae* serovar 1,13,23:z:1,5 HAZEL-067 |
| HAZEL-068 | PRJNA602741 | SAMN13905978 | JAAHQK000000000 | *Salmonella enterica* subsp. *enterica* serovar Karamoja HAZEL-068 |
| HAZEL-069 | PRJNA602741 | SAMN13905979 | JAAHQJ000000000 | *Salmonella enterica* subsp. *enterica* serovar Karamoja HAZEL-069 |
| HAZEL-070 | PRJNA602741 | SAMN13905980 | JAAHUF000000000 | *Salmonella enterica* subsp. *salamae* serovar 1,13,23:z:1,5 HAZEL-070 |
| HAZEL-071 | PRJNA602741 | SAMN13905981 | JAAHUE000000000 | *Salmonella enterica* subsp. *salamae* serovar 1,13,23:z:1,5 HAZEL-071 |
| HAZEL-072 | PRJNA602741 | SAMN13905982 | JAAHUD000000000 | *Salmonella enterica* subsp. *salamae* serovar 1,13,23:z:1,5 HAZEL-072 |
| HAZEL-073 | PRJNA602741 | SAMN13905983 | JAAHQI000000000 | *Salmonella enterica* subsp. *enterica* serovar Karamoja HAZEL-073 |
| HAZEL-074 | PRJNA602741 | SAMN13905984 | JAAHQH000000000 | *Salmonella enterica* subsp. *enterica* serovar Give HAZEL-074 |
| HAZEL-076 | PRJNA602741 | SAMN13905985 | JAAHQG000000000 | *Salmonella enterica* subsp. *enterica* serovar Give HAZEL-076 |
| HAZEL-077 | PRJNA602741 | SAMN13905986 | JAAHQF000000000 | *Salmonella enterica* subsp. *enterica* serovar Give HAZEL-077 |
| HAZEL-078 | PRJNA602741 | SAMN13905987 | JAAHQE000000000 | *Salmonella enterica* subsp. *enterica* HAZEL-078 |
| HAZEL-079 | PRJNA602741 | SAMN13905988 | JAAHQD000000000 | *Salmonella enterica* subsp. *enterica* HAZEL-079 |
| HAZEL-080 | PRJNA602741 | SAMN13905989 | JAAHQC000000000 | *Salmonella enterica* subsp. *salamae* serovar 42:r:- HAZEL-080 |
| HAZEL-081 | PRJNA602741 | SAMN13905990 | JAAHQB000000000 | *Salmonella enterica* subsp. *salamae* serovar 42:r:- HAZEL-081 |
| HAZEL-082 | PRJNA602741 | SAMN13905991 | JAAHQA000000000 | *Salmonella enterica* subsp. *enterica* serovar Enteritidis HAZEL-082 |
| HAZEL-083 | PRJNA602741 | SAMN13905992 | JAAHPZ000000000 | *Salmonella enterica* subsp. *enterica* serovar Enteritidis HAZEL-083 |
| HAZEL-084 | PRJNA602741 | SAMN13905993 | JAAHPY000000000 | *Salmonella enterica* subsp. *enterica* serovar Hadar HAZEL-084 |
| HAZEL-085 | PRJNA602741 | SAMN13905994 | JAAHPX000000000 | *Salmonella enterica* subsp. *enterica* serovar Hadar HAZEL-085 |
| HAZEL-086 | PRJNA602741 | SAMN13905995 | JAAHPW000000000 | *Salmonella enterica* subsp. *enterica* serovar Orion HAZEL-086 |
| HAZEL-087 | PRJNA602741 | SAMN13905996 | JAAHPV000000000 | *Salmonella enterica* subsp. *enterica* serovar Orion HAZEL-087 |
| HAZEL-088 | PRJNA602741 | SAMN13905997 | JAAHPU000000000 | *Salmonella enterica* subsp. *enterica* serovar Orion HAZEL-088 |
| HAZEL-089 | PRJNA602741 | SAMN13905998 | JAAHPT000000000 | *Salmonella enterica* subsp. *enterica* serovar Orion HAZEL-089 |
| HAZEL-090 | PRJNA602741 | SAMN13905999 | JAAHPS000000000 | *Salmonella enterica* subsp. *enterica* serovar Orion HAZEL-090 |
| HAZEL-091 | PRJNA602741 | SAMN13906000 | JAAHPR000000000 | *Salmonella enterica* subsp. *enterica* serovar Orion HAZEL-091 |
| HAZEL-092 | PRJNA602741 | SAMN13906001 | JAAHPQ000000000 | *Salmonella enterica* subsp. *enterica* serovar Orion HAZEL-092 |
| HAZEL-093 | PRJNA602741 | SAMN13906002 | JAAHPP000000000 | *Salmonella enterica* subsp. *enterica* serovar Orion HAZEL-093 |
| HAZEL-094 | PRJNA602741 | SAMN13906003 | JAAHUC000000000 | *Salmonella enterica* subsp. *enterica* serovar 42:r:- HAZEL-094 |
| HAZEL-095 | PRJNA602741 | SAMN13906004 | JAAHPO000000000 | *Salmonella enterica* subsp. *salamae* serovar 42:r:- HAZEL-095 |
| HAZEL-096 | PRJNA602741 | SAMN13906005 | JAAHPN000000000 | *Salmonella enterica* subsp. *enterica* serovar Saintpaul HAZEL-096 |
| HAZEL-097 | PRJNA602741 | SAMN13906006 | JAAHPM000000000 | *Salmonella enterica* subsp. *enterica* serovar Saintpaul HAZEL-097 |
| HAZEL-098 | PRJNA602741 | SAMN13906007 | JAAHPL000000000 | *Salmonella enterica* subsp. *enterica* serovar Saintpaul HAZEL-098 |
| HAZEL-099 | PRJNA602741 | SAMN13906008 | JAAHPK000000000 | *Salmonella enterica* subsp. *enterica* serovar Saintpaul HAZEL-099 |
| HAZEL-100 | PRJNA602741 | SAMN13906009 | JAAHPJ000000000 | *Salmonella enterica* subsp. *enterica* serovar Karamoja HAZEL-100 |
| HAZEL-101 | PRJNA602741 | SAMN13906010 | JAAHPI000000000 | *Salmonella enterica* subsp. *enterica* serovar Karamoja HAZEL-101 |
| HAZEL-102 | PRJNA602741 | SAMN13906011 | JAAHPH000000000 | *Salmonella enterica* subsp. *enterica* serovar Jangwani HAZEL-102 |
| HAZEL-103 | PRJNA602741 | SAMN13906012 | JAAHPG000000000 | *Salmonella enterica* subsp. *enterica* serovar Jangwani HAZEL-103 |
| HAZEL-104 | PRJNA602741 | SAMN13906013 | JAAHPF000000000 | *Salmonella enterica* subsp. *enterica* serovar Jangwani HAZEL-104 |
| HAZEL-105 | PRJNA602741 | SAMN13906014 | JAAHPE000000000 | *Salmonella enterica* subsp. *enterica* serovar Jangwani HAZEL-105 |
| HAZEL-106 | PRJNA602741 | SAMN13906015 | JAAHPD000000000 | *Salmonella enterica* subsp. *enterica* serovar Saintpaul HAZEL-106 |
| HAZEL-107 | PRJNA602741 | SAMN13906016 | JAAHPC000000000 | *Salmonella enterica* subsp. *enterica* serovar Saintpaul HAZEL-107 |
| HAZEL-108 | PRJNA602741 | SAMN13906017 | JAAHPB000000000 | *Salmonella enterica* subsp. *enterica* serovar Saintpaul HAZEL-108 |
| HAZEL-109 | PRJNA602741 | SAMN13906018 | JAAHPA000000000 | *Salmonella enterica* subsp. *enterica* serovar Orion HAZEL-109 |
| HAZEL-110 | PRJNA602741 | SAMN13906019 | JAAHOZ000000000 | *Salmonella enterica* subsp. *enterica* serovar Orion HAZEL-110 |
| HAZEL-111 | PRJNA602741 | SAMN13906020 | JAAHOY000000000 | *Salmonella enterica* subsp. *enterica* serovar Heidelberg HAZEL-111 |
| HAZEL-112 | PRJNA602741 | SAMN13906021 | JAAHSX000000000 | *Salmonella enterica* subsp. *enterica* serovar Heidelberg HAZEL-112 |
| HAZEL-113 | PRJNA602741 | SAMN13906022 | JAAHOX000000000 | *Salmonella enterica* subsp. *salamae* serovar 42:r:- HAZEL-113 |
| HAZEL-114 | PRJNA602741 | SAMN13906023 | JAAHOW000000000 | *Salmonella enterica* subsp. *enterica* serovar Hvittingfoss HAZEL-114 |
| HAZEL-115 | PRJNA602741 | SAMN13906024 | JAAHOV000000000 | *Salmonella enterica* subsp. *enterica* serovar Hvittingfoss HAZEL-115 |
| HAZEL-116 | PRJNA602741 | SAMN13906025 | JAAHOU000000000 | *Salmonella enterica* subsp. *enterica* serovar Livingstone HAZEL-116 |
| HAZEL-117 | PRJNA602741 | SAMN13906026 | JAAHOT000000000 | *Salmonella enterica* subsp. *enterica* serovar Livingstone HAZEL-117 |
| HAZEL-118 | PRJNA602741 | SAMN13906027 | JAAHOS000000000 | *Salmonella enterica* subsp. *enterica* serovar Livingstone HAZEL-118 |
| HAZEL-119 | PRJNA602741 | SAMN13906028 | JAAHOR000000000 | *Salmonella enterica* subsp. *salamae* serovar 42:r:- HAZEL-119 |
| HAZEL-120 | PRJNA602741 | SAMN13906029 | JAAHOQ000000000 | *Salmonella enterica* subsp. *salamae* serovar 42:r:- HAZEL-120 |
| HAZEL-121 | PRJNA602741 | SAMN13906030 | JAAHOP000000000 | *Salmonella enterica* subsp. *enterica* serovar Kibusi HAZEL-121 |
| HAZEL-122 | PRJNA602741 | SAMN13906031 | JAAHOO000000000 | *Salmonella enterica* subsp. *enterica* serovar Kibusi HAZEL-122 |
| HAZEL-123 | PRJNA602741 | SAMN13906032 | JAAHON000000000 | *Salmonella enterica* subsp. *enterica* serovar Kibusi HAZEL-123 |
| HAZEL-124 | PRJNA602741 | SAMN13906033 | JAAHOM000000000 | *Salmonella enterica* subsp. *enterica* serovar Kibusi HAZEL-124 |
| HAZEL-125 | PRJNA602741 | SAMN13906034 | JAAHOL000000000 | *Salmonella enterica* subsp. *enterica* serovar Kibusi HAZEL-125 |
| HAZEL-126 | PRJNA602741 | SAMN13906035 | JAAHOK000000000 | *Salmonella enterica* subsp. *enterica* serovar Kibusi HAZEL-126 |
| HAZEL-127 | PRJNA602741 | SAMN13906036 | JAAHOJ000000000 | *Salmonella enterica* subsp. *enterica* serovar Kibusi HAZEL-127 |
| HAZEL-128 | PRJNA602741 | SAMN13906037 | JAAHOI000000000 | *Salmonella enterica* subsp. *enterica* serovar Kibusi HAZEL-128 |
| HAZEL-129 | PRJNA602741 | SAMN13906038 | JAAHOH000000000 | *Salmonella enterica* subsp. *enterica* serovar Jangwani HAZEL-129 |
| HAZEL-130 | PRJNA602741 | SAMN13906039 | JAAHOG000000000 | *Salmonella enterica* subsp. *enterica* serovar Jangwani HAZEL-130 |
| HAZEL-131 | PRJNA602741 | SAMN13906040 | JAAHOF000000000 | *Salmonella enterica* subsp. *enterica* serovar Onderstepoort HAZEL-131 |
| HAZEL-132 | PRJNA602741 | SAMN13906041 | JAAHOE000000000 | *Salmonella enterica* subsp. *enterica* serovar Onderstepoort HAZEL-132 |
| HAZEL-133 | PRJNA602741 | SAMN13906042 | JAAHOD000000000 | *Salmonella enterica* subsp. *enterica* serovar Typhimurium HAZEL-133 |
| HAZEL-134 | PRJNA602741 | SAMN13906043 | JAAHOC000000000 | *Salmonella enterica* subsp. *enterica* serovar Kibusi HAZEL-134 |
| HAZEL-135 | PRJNA602741 | SAMN13906044 | JAAHOB000000000 | *Salmonella enterica* subsp. *enterica* serovar Umbilo HAZEL-135 |
| HAZEL-136 | PRJNA602741 | SAMN13906045 | JAAHOA000000000 | *Salmonella enterica* subsp. *enterica* serovar Umbilo HAZEL-136 |
| HAZEL-137 | PRJNA602741 | SAMN13906046 | JAAHNZ000000000 | *Salmonella enterica* subsp. *enterica* serovar Saintpaul HAZEL-137 |
| HAZEL-138 | PRJNA602741 | SAMN13906047 | JAAHNY000000000 | *Salmonella enterica* subsp. *enterica* serovar Saintpaul HAZEL-138 |
| HAZEL-139 | PRJNA602741 | SAMN13906048 | JAAHNX000000000 | *Salmonella enterica* subsp. *enterica* serovar Typhimurium HAZEL-139 |
| HAZEL-140 | PRJNA602741 | SAMN13906049 | JAAHNW000000000 | *Salmonella enterica* subsp. *enterica* serovar Typhimurium HAZEL-140 |
| HAZEL-141 | PRJNA602741 | SAMN13906050 | JAAHNV000000000 | *Salmonella enterica* subsp. *enterica* serovar Richmond HAZEL-141 |
| HAZEL-142 | PRJNA602741 | SAMN13906051 | JAAHNU000000000 | *Salmonella enterica* subsp. *enterica* serovar Saintpaul HAZEL-142 |
| HAZEL-143 | PRJNA602741 | SAMN13906052 | JAAHNT000000000 | *Salmonella enterica* subsp. *enterica* serovar Orion HAZEL-143 |
| HAZEL-144 | PRJNA602741 | SAMN13906053 | JAAHNS000000000 | *Salmonella enterica* subsp. *enterica* serovar Orion HAZEL-144 |
| HAZEL-145 | PRJNA602741 | SAMN13906054 | JAAHNR000000000 | *Salmonella enterica* subsp. *enterica* serovar Orion HAZEL-145 |
| HAZEL-146 | PRJNA602741 | SAMN13906055 | JAAHNQ000000000 | *Salmonella enterica* subsp. *enterica* serovar Orion HAZEL-146 |
| HAZEL-147 | PRJNA602741 | SAMN13906056 | JAAHNP000000000 | *Salmonella enterica* subsp. *enterica* serovar Indiana HAZEL-147 |
| HAZEL-148 | PRJNA602741 | SAMN13906057 | JAAHNO000000000 | *Salmonella enterica* subsp. *enterica* serovar Indiana HAZEL-148 |
| HAZEL-149 | PRJNA602741 | SAMN13906058 | JAAHNN000000000 | *Salmonella enterica* subsp. *enterica* serovar Orion HAZEL-149 |
| HAZEL-150 | PRJNA602741 | SAMN13906059 | JAAHSZ000000000 | *Salmonella enterica* subsp. *enterica* serovar Orion HAZEL-150 |
| HAZEL-151 | PRJNA602741 | SAMN13906060 | JAAHNM000000000 | *Salmonella enterica* subsp. *enterica* serovar Durban HAZEL-151 |
| HAZEL-152 | PRJNA602741 | SAMN13906061 | JAAHNL000000000 | *Salmonella enterica* subsp. *enterica* serovar Durban HAZEL-152 |
| HAZEL-153 | PRJNA602741 | SAMN13906062 | JAAHNK000000000 | *Salmonella enterica* subsp. *enterica* serovar Eastbourne HAZEL-153 |
| HAZEL-154 | PRJNA602741 | SAMN13906063 | JAAHNJ000000000 | *Salmonella enterica* subsp. *enterica* serovar Kingabwa HAZEL-154 |
| HAZEL-155 | PRJNA602741 | SAMN13906064 | JAAHNI000000000 | *Salmonella enterica* subsp. *enterica* serovar Agona HAZEL-155 |
| HAZEL-156 | PRJNA602741 | SAMN13906065 | JAAHNH000000000 | *Salmonella enterica* subsp. *enterica* serovar Kentucky HAZEL-156 |
| HAZEL-157 | PRJNA602741 | SAMN13906066 | JAAHNG000000000 | *Salmonella enterica* subsp. *enterica* serovar Kentucky HAZEL-157 |
| HAZEL-158 | PRJNA602741 | SAMN13906067 | JAAHNF000000000 | *Salmonella enterica* subsp. *enterica* HAZEL-158 |
| HAZEL-159 | PRJNA602741 | SAMN13906068 | JAAHNE000000000 | *Salmonella enterica* subsp. *enterica* HAZEL-159 |
| HAZEL-160 | PRJNA602741 | SAMN13906069 | JAAHND000000000 | *Salmonella enterica* subsp. *enterica* serovar Kisarawe HAZEL-160 |
| HAZEL-161 | PRJNA602741 | SAMN13906070 | JAAHNC000000000 | *Salmonella enterica* subsp. *enterica* serovar Kisarawe HAZEL-161 |
| HAZEL-162 | PRJNA602741 | SAMN13906071 | JAAHNB000000000 | *Salmonella enterica* subsp. *enterica* serovar Kisarawe HAZEL-162 |
| HAZEL-163 | PRJNA602741 | SAMN13906072 | JAAHNA000000000 | *Salmonella enterica* subsp. *enterica* serovar Kisarawe HAZEL-163 |
| HAZEL-164 | PRJNA602741 | SAMN13906073 | JAAHMZ000000000 | *Salmonella enterica* subsp. *enterica* serovar Heidelberg HAZEL-164 |
| HAZEL-165 | PRJNA602741 | SAMN13906074 | JAAHMY000000000 | *Salmonella enterica* subsp. *enterica* serovar Heidelberg HAZEL-165 |
| HAZEL-166 | PRJNA602741 | SAMN13906075 | JAAHMX000000000 | *Salmonella enterica* subsp. *enterica* serovar Jangwani HAZEL-166 |
| HAZEL-167 | PRJNA602741 | SAMN13906076 | JAAHMW000000000 | *Salmonella enterica* subsp. *enterica* serovar Jangwani HAZEL-167 |
| HAZEL-168 | PRJNA602741 | SAMN13906077 | JAAHMV000000000 | *Salmonella enterica* subsp. *enterica* serovar Orion HAZEL-168 |
| HAZEL-173 | PRJNA602741 | SAMN13906078 | JAAHMU000000000 | *Salmonella enterica* subsp. *enterica* serovar Enteritidis HAZEL-173 |
| HAZEL-174 | PRJNA602741 | SAMN13906079 | JAAHMT000000000 | *Salmonella enterica* subsp. *enterica* serovar Enteritidis HAZEL-174 |
| HAZEL-175 | PRJNA602741 | SAMN13906080 | JAAHMS000000000 | *Salmonella enterica* subsp. *salamae* serovar 42:r:- HAZEL-175 |
| HAZEL-176 | PRJNA602741 | SAMN13906081 | JAAHMR000000000 | *Salmonella enterica* subsp. *salamae* serovar 42:r:- HAZEL-176 |
| HAZEL-177 | PRJNA602741 | SAMN13906082 | JAAHMQ000000000 | *Salmonella enterica* subsp. *salamae* serovar 42:r:- HAZEL-177 |
| HAZEL-178 | PRJNA602741 | SAMN13906083 | JAAHMP000000000 | *Salmonella enterica* subsp. *salamae* serovar 42:r:- HAZEL-178 |
| HAZEL-179 | PRJNA602741 | SAMN13906084 | JAAHMO000000000 | *Salmonella enterica* subsp. *enterica* serovar Typhimurium HAZEL-179 |
| HAZEL-180 | PRJNA602741 | SAMN13906085 | JAAHMN000000000 | *Salmonella enterica* subsp. *enterica* serovar Agona HAZEL-180 |
| HAZEL-181 | PRJNA602741 | SAMN13906086 | JAAHMM000000000 | *Salmonella enterica* subsp. *enterica* serovar Kentucky HAZEL-181 |
| HAZEL-182 | PRJNA602741 | SAMN13906087 | JAAHML000000000 | *Salmonella enterica* subsp. *enterica* serovar Eastbourne HAZEL-182 |
| HAZEL-183 | PRJNA602741 | SAMN13906088 | JAAHMK000000000 | *Salmonella enterica* subsp. *enterica* serovar Eastbourne HAZEL-183 |
| HAZEL-184 | PRJNA602741 | SAMN13906089 | JAAHMJ000000000 | *Salmonella enterica* subsp. *enterica* serovar Eastbourne HAZEL-184 |
| HAZEL-185 | PRJNA602741 | SAMN13906090 | JAAHMI000000000 | *Salmonella enterica* subsp. *enterica* HAZEL-185 |
| HAZEL-186 | PRJNA602741 | SAMN13906091 | JAAHMH000000000 | *Salmonella enterica* subsp. *enterica* HAZEL-186 |
| HAZEL-187 | PRJNA602741 | SAMN13906092 | JAAHMG000000000 | *Salmonella enterica* subsp. *enterica* serovar Saintpaul HAZEL-187 |
| HAZEL-188 | PRJNA602741 | SAMN13906093 | JAAHMF000000000 | *Salmonella enterica* subsp. *enterica* serovar Saintpaul HAZEL-188 |
| HAZEL-189 | PRJNA602741 | SAMN13906094 | JAAHME000000000 | *Salmonella enterica* subsp. *enterica* serovar Aberdeen HAZEL-189 |
| HAZEL-190 | PRJNA602741 | SAMN13906095 | JAAHMD000000000 | *Salmonella enterica* subsp. *enterica* serovar Aberdeen HAZEL-190 |
| HAZEL-191 | PRJNA602741 | SAMN13906096 | JAAHMC000000000 | *Salmonella enterica* subsp. *enterica* serovar Leoben HAZEL-191 |
| HAZEL-192 | PRJNA602741 | SAMN13906097 | JAAHMB000000000 | *Salmonella enterica* subsp. *enterica* serovar Leoben HAZEL-192 |
| HAZEL-193 | PRJNA602741 | SAMN13906098 | JAAHMA000000000 | *Salmonella enterica* subsp. *enterica* serovar Breda HAZEL-193 |
| HAZEL-194 | PRJNA602741 | SAMN13906099 | JAAHLZ000000000 | *Salmonella enterica* subsp. *enterica* serovar Breda HAZEL-194 |
| HAZEL-195 | PRJNA602741 | SAMN13906100 | JAAHUB000000000 | *Salmonella enterica* subsp. *salamae* serovar 1,13,23:z29:e,n,x HAZEL-195 |
| HAZEL-196 | PRJNA602741 | SAMN13906101 | JAAHUA000000000 | *Salmonella enterica* subsp. *salamae* serovar 1,13,23:z29:e,n,x HAZEL-196 |
| HAZEL-197 | PRJNA602741 | SAMN13906102 | JAAHTZ000000000 | *Salmonella enterica* subsp. *salamae* serovar 1,13,23:z29:e,n,x HAZEL-197 |
| HAZEL-198 | PRJNA602741 | SAMN13906103 | JAAHTY000000000 | *Salmonella enterica* subsp. *salamae* serovar 1,13,23:z29:e,n,x HAZEL-198 |
| HAZEL-199 | PRJNA602741 | SAMN13906104 | JAAHTX000000000 | *Salmonella enterica* subsp. *salamae* serovar 1,13,23:z29:e,n,x HAZEL-199 |
| HAZEL-200 | PRJNA602741 | SAMN13906105 | JAAHLY000000000 | *Salmonella enterica* subsp. *enterica* serovar Karamoja HAZEL-200 |
| HAZEL-201 | PRJNA602741 | SAMN13906106 | JAAHLX000000000 | *Salmonella enterica* subsp. *enterica* serovar Karamoja HAZEL-201 |
| HAZEL-202 | PRJNA602741 | SAMN13906107 | JAAHTW000000000 | *Salmonella enterica* subsp. *salamae* serovar 1,13,23:z:1,5 HAZEL-202 |
| HAZEL-203 | PRJNA602741 | SAMN13906108 | JAAHTV000000000 | *Salmonella enterica* subsp. *salamae* serovar 1,13,23:z:1,5 HAZEL-203 |
| HAZEL-204 | PRJNA602741 | SAMN13906109 | JAAHLW000000000 | *Salmonella enterica* subsp. *enterica* serovar Karamoja HAZEL-204 |
| HAZEL-205 | PRJNA602741 | SAMN13906110 | JAAHLV000000000 | *Salmonella enterica* subsp. *enterica* serovar Karamoja HAZEL-205 |
| HAZEL-206 | PRJNA602741 | SAMN13906111 | JAAHLU000000000 | *Salmonella enterica* subsp. *enterica* serovar Karamoja HAZEL-206 |
| HAZEL-207 | PRJNA602741 | SAMN13906112 | JAAHLT000000000 | *Salmonella enterica* subsp. *enterica* serovar Karamoja HAZEL-207 |
| HAZEL-208 | PRJNA602741 | SAMN13906113 | JAAHLS000000000 | *Salmonella enterica* subsp. *enterica* serovar Karamoja HAZEL-208 |
| HAZEL-209 | PRJNA602741 | SAMN13906114 | JAAHLR000000000 | *Salmonella enterica* subsp. *enterica* serovar Epinay HAZEL-209 |
| HAZEL-210 | PRJNA602741 | SAMN13906115 | JAAHLQ000000000 | *Salmonella enterica* subsp. *enterica* serovar Epinay HAZEL-210 |
| HAZEL-211 | PRJNA602741 | SAMN13906116 | JAAHTU000000000 | *Salmonella enterica* subsp. *salamae* serovar 1,9,12,46,27:l,w:e,n,x HAZEL-211 |
| HAZEL-212 | PRJNA602741 | SAMN13906117 | JAAHTT000000000 | *Salmonella enterica* subsp. *salamae* serovar 1,9,12,46,27:l,w:e,n,x HAZEL-212 |
| HAZEL-213 | PRJNA602741 | SAMN13906118 | JAAHTS000000000 | *Salmonella enterica* subsp. *salamae* serovar 1,9,12,46,27:l,w:e,n,x HAZEL-213 |
| HAZEL-214 | PRJNA602741 | SAMN13906119 | JAAHTR000000000 | *Salmonella enterica* subsp. *salamae* serovar 1,9,12,46,27:l,w:e,n,x HAZEL-214 |
| HAZEL-215 | PRJNA602741 | SAMN13906120 | JAAHLP000000000 | *Salmonella enterica* subsp. *enterica* serovar Orion HAZEL-215 |
| HAZEL-216 | PRJNA602741 | SAMN13906121 | JAAHLO000000000 | *Salmonella enterica* subsp. *enterica* serovar Orion HAZEL-216 |
| HAZEL-217 | PRJNA602741 | SAMN13906122 | JAAHLN000000000 | *Salmonella enterica* subsp. *enterica* serovar Orion HAZEL-217 |
| HAZEL-218 | PRJNA602741 | SAMN13906123 | JAAHLM000000000 | *Salmonella enterica* subsp. *enterica* serovar Orion HAZEL-218 |
| HAZEL-219 | PRJNA602741 | SAMN13906124 | JAAHLL000000000 | *Salmonella enterica* subsp. *enterica* serovar Orion HAZEL-219 |
| HAZEL-220 | PRJNA602741 | SAMN13906125 | JAAHLK000000000 | *Salmonella enterica* subsp. *enterica* serovar Typhimurium HAZEL-220 |
| HAZEL-221 | PRJNA602741 | SAMN13906126 | JAAHLJ000000000 | *Salmonella enterica* subsp. *enterica* serovar Typhimurium HAZEL-221 |
| HAZEL-222 | PRJNA602741 | SAMN13906127 | JAAHLI000000000 | *Salmonella enterica* subsp. *enterica* serovar Typhimurium HAZEL-222 |
| HAZEL-223 | PRJNA602741 | SAMN13906128 | JAAHLH000000000 | *Salmonella enterica* subsp. *enterica* serovar Typhimurium HAZEL-223 |
| HAZEL-224 | PRJNA602741 | SAMN13906129 | JAAHLG000000000 | *Salmonella enterica* subsp. *enterica* serovar Typhimurium HAZEL-224 |
| HAZEL-225 | PRJNA602741 | SAMN13906130 | JAAHLF000000000 | *Salmonella enterica* subsp. *salamae* serovar 42:r:- HAZEL-225 |
| HAZEL-226 | PRJNA602741 | SAMN13906131 | JAAHLE000000000 | *Salmonella enterica* subsp. *enterica* serovar Kiambu HAZEL-226 |
| HAZEL-227 | PRJNA602741 | SAMN13906132 | JAAHLD000000000 | *Salmonella enterica* subsp. *enterica* serovar Kisarawe HAZEL-227 |
| HAZEL-228 | PRJNA602741 | SAMN13906133 | JAAHTQ000000000 | *Salmonella enterica* subsp. *salamae* serovar 1,4,12,27:e,n,x:e,n,x HAZEL-228 |
| HAZEL-229 | PRJNA602741 | SAMN13906134 | JAAHLC000000000 | *Salmonella enterica* subsp. *enterica* serovar Kisarawe HAZEL-229 |
| HAZEL-230 | PRJNA602741 | SAMN13906135 | JAAHTP000000000 | *Salmonella enterica* subsp. *salamae* serovar 1,4,12,27:e,n,x:e,n,x HAZEL-230 |
| HAZEL-231 | PRJNA602741 | SAMN13906136 | JAAHLB000000000 | *Salmonella enterica* subsp. *enterica* serovar Senftenberg HAZEL-231 |
| HAZEL-232 | PRJNA602741 | SAMN13906137 | JAAHLA000000000 | *Salmonella enterica* subsp. *enterica* serovar Senftenberg HAZEL-232 |
| HAZEL-233 | PRJNA602741 | SAMN13906138 | JAAHKZ000000000 | *Salmonella enterica* subsp. *enterica* serovar Agona HAZEL-233 |
| HAZEL-234 | PRJNA602741 | SAMN13906139 | JAAHKY000000000 | *Salmonella enterica* subsp. *enterica* serovar Agona HAZEL-234 |
| HAZEL-237 | PRJNA602741 | SAMN13906140 | JAAHTO000000000 | *Salmonella enterica* subsp. *salamae* serovar 1,4,12,27:e,n,x:e,n,x HAZEL-237 |
| HAZEL-238 | PRJNA602741 | SAMN13906141 | JAAHTN000000000 | *Salmonella enterica* subsp. *salamae* serovar 1,4,12,27:e,n,x:e,n,x HAZEL-238 |
| HAZEL-239 | PRJNA602741 | SAMN13906142 | JAAHKX000000000 | *Salmonella enterica* subsp. *enterica* serovar Saintpaul HAZEL-239 |
| HAZEL-240 | PRJNA602741 | SAMN13906143 | JAAHKW000000000 | *Salmonella enterica* subsp. *enterica* serovar Newport HAZEL-240 |
| HAZEL-241 | PRJNA602741 | SAMN13906144 | JAAHKV000000000 | *Salmonella enterica* subsp. *enterica* serovar Eastbourne HAZEL-241 |
| HAZEL-242 | PRJNA602741 | SAMN13906145 | JAAHKU000000000 | *Salmonella enterica* subsp. *enterica* serovar Eastbourne HAZEL-242 |
| HAZEL-243 | PRJNA602741 | SAMN13906146 | JAAHKT000000000 | *Salmonella enterica* subsp. *enterica* serovar Agona HAZEL-243 |
| HAZEL-244 | PRJNA602741 | SAMN13906147 | JAAHKS000000000 | *Salmonella enterica* subsp. *enterica* serovar Kentucky HAZEL-244 |
| HAZEL-245 | PRJNA602741 | SAMN13906148 | JAAHKR000000000 | *Salmonella enterica* subsp. *enterica* serovar Yarrabah HAZEL-245 |
| HAZEL-246 | PRJNA602741 | SAMN13906149 | JAAHKQ000000000 | *Salmonella enterica* subsp. *enterica* serovar Yarrabah HAZEL-246 |
| HAZEL-247 | PRJNA602741 | SAMN13906150 | JAAHKP000000000 | *Salmonella enterica* subsp. *salamae* HAZEL-247 |
| HAZEL-248 | PRJNA602741 | SAMN13906151 | JAAHKO000000000 | *Salmonella enterica* subsp. *salamae* HAZEL-248 |
| HAZEL-249 | PRJNA602741 | SAMN13906152 | JAAHKN000000000 | *Salmonella enterica* subsp. *enterica* serovar Braenderup HAZEL-249 |
| HAZEL-250 | PRJNA602741 | SAMN13906153 | JAAHKM000000000 | *Salmonella enterica* subsp. *enterica* serovar Braenderup HAZEL-250 |
| HAZEL-251 | PRJNA602741 | SAMN13906154 | JAAHKL000000000 | *Salmonella enterica* subsp. *enterica* serovar Saintpaul HAZEL-251 |
| HAZEL-252 | PRJNA602741 | SAMN13906155 | JAAHKK000000000 | *Salmonella enterica* subsp. *enterica* serovar Kisarawe HAZEL-252 |
| HAZEL-253 | PRJNA602741 | SAMN13906156 | JAAHKJ000000000 | *Salmonella enterica* subsp. *enterica* serovar Orion HAZEL-253 |
| HAZEL-254 | PRJNA602741 | SAMN13906157 | JAAHKI000000000 | *Salmonella enterica* subsp. *enterica* serovar Orion HAZEL-254 |
| HAZEL-255 | PRJNA602741 | SAMN13906158 | JAAHKH000000000 | *Salmonella enterica* subsp. *enterica* serovar Poona HAZEL-255 |
| HAZEL-256 | PRJNA602741 | SAMN13906159 | JAAHKG000000000 | *Salmonella enterica* subsp. *enterica* serovar Jangwani HAZEL-256 |
| HAZEL-257 | PRJNA602741 | SAMN13906160 | JAAHKF000000000 | *Salmonella enterica* subsp. *enterica* serovar Jangwani HAZEL-257 |
| HAZEL-258 | PRJNA602741 | SAMN13906161 | JAAHKE000000000 | *Salmonella enterica* subsp. *enterica* serovar Jangwani HAZEL-258 |
| HAZEL-259 | PRJNA602741 | SAMN13906162 | JAAHKD000000000 | *Salmonella enterica* subsp. *enterica* serovar Enteritidis HAZEL-259 |
| HAZEL-260 | PRJNA602741 | SAMN13906163 | JAAHKC000000000 | *Salmonella enterica* subsp. *enterica* serovar Enteritidis HAZEL-260 |
| HAZEL-261 | PRJNA602741 | SAMN13906164 | JAAHKB000000000 | *Salmonella enterica* subsp. *salamae* HAZEL-261 |
| HAZEL-262 | PRJNA602741 | SAMN13906165 | JAAHKA000000000 | *Salmonella enterica* subsp. *salamae* HAZEL-262 |
| HAZEL-263 | PRJNA602741 | SAMN13906166 | JAAHJZ000000000 | *Salmonella enterica* subsp. *enterica* serovar Enteritidis HAZEL-263 |
| HAZEL-264 | PRJNA602741 | SAMN13906167 | JAAHJY000000000 | *Salmonella enterica* subsp. *enterica* serovar Enteritidis HAZEL-264 |
| HAZEL-265 | PRJNA602741 | SAMN13906168 | JAAHJX000000000 | *Salmonella enterica* subsp. *enterica* serovar Enteritidis HAZEL-265 |
| HAZEL-266 | PRJNA602741 | SAMN13906169 | JAAHJW000000000 | *Salmonella enterica* subsp. *enterica* serovar Enteritidis HAZEL-266 |
| HAZEL-267 | PRJNA602741 | SAMN13906170 | JAAHJV000000000 | *Salmonella enterica* subsp. *enterica* serovar Enteritidis HAZEL-267 |
| HAZEL-268 | PRJNA602741 | SAMN13906171 | JAAHJU000000000 | *Salmonella enterica* subsp. *enterica* serovar Newport HAZEL-268 |
| HAZEL-269 | PRJNA602741 | SAMN13906172 | JAAHJT000000000 | *Salmonella enterica* subsp. *enterica* serovar Newport HAZEL-269 |
| HAZEL-270 | PRJNA602741 | SAMN13906173 | JAAHJS000000000 | *Salmonella enterica* subsp. *enterica* serovar Newport HAZEL-270 |
| HAZEL-271 | PRJNA602741 | SAMN13906174 | JAAHJR000000000 | *Salmonella enterica* subsp. *enterica* serovar Newport HAZEL-271 |
| HAZEL-272 | PRJNA602741 | SAMN13906175 | JAAHJQ000000000 | *Salmonella enterica* subsp. *enterica* serovar Muenchen HAZEL-272 |
| HAZEL-273 | PRJNA602741 | SAMN13906176 | JAAHJP000000000 | *Salmonella enterica* subsp. *enterica* serovar Muenchen HAZEL-273 |
| HAZEL-274 | PRJNA602741 | SAMN13906177 | JAAHJO000000000 | *Salmonella enterica* subsp. *enterica* serovar Virchow HAZEL-274 |
| HAZEL-275 | PRJNA602741 | SAMN13906178 | JAAHJN000000000 | *Salmonella enterica* subsp. *enterica* serovar Virchow HAZEL-275 |
| HAZEL-276 | PRJNA602741 | SAMN13906179 | JAAHJM000000000 | *Salmonella enterica* subsp. *enterica* serovar Kentucky HAZEL-276 |
| HAZEL-277 | PRJNA602741 | SAMN13906180 | JAAHJL000000000 | *Salmonella enterica* subsp. *enterica* serovar Kentucky HAZEL-277 |
| HAZEL-278 | PRJNA602741 | SAMN13906181 | JAAHJK000000000 | *Salmonella enterica* subsp. *enterica* serovar Kentucky HAZEL-278 |
| HAZEL-279 | PRJNA602741 | SAMN13906182 | JAAHJJ000000000 | *Salmonella enterica* subsp. *enterica* serovar Enteritidis HAZEL-279 |
| HAZEL-280 | PRJNA602741 | SAMN13906183 | JAAHJI000000000 | *Salmonella enterica* subsp. *enterica* serovar Enteritidis HAZEL-280 |
| HAZEL-281 | PRJNA602741 | SAMN13906184 | JAAHJH000000000 | *Salmonella enterica* subsp. *enterica* serovar Enteritidis HAZEL-281 |
| HAZEL-282 | PRJNA602741 | SAMN13906185 | JAAHJG000000000 | *Salmonella enterica* subsp. *enterica* serovar Enteritidis HAZEL-282 |
| HAZEL-283 | PRJNA602741 | SAMN13906186 | JAAHJF000000000 | *Salmonella enterica* subsp. *enterica* serovar Newport HAZEL-283 |
| HAZEL-284 | PRJNA602741 | SAMN13906187 | JAAHJE000000000 | *Salmonella enterica* subsp. *enterica* serovar Newport HAZEL-284 |
| HAZEL-285 | PRJNA602741 | SAMN13906188 | JAAHJD000000000 | *Salmonella enterica* subsp. *enterica* serovar Newport HAZEL-285 |
| HAZEL-286 | PRJNA602741 | SAMN13906189 | JAAHJC000000000 | *Salmonella enterica* subsp. *enterica* serovar Newport HAZEL-286 |
| HAZEL-287 | PRJNA602741 | SAMN13906190 | JAAHJB000000000 | *Salmonella enterica* subsp. *enterica* serovar Newport HAZEL-287 |
| HAZEL-288 | PRJNA602741 | SAMN13906191 | JAAHJA000000000 | *Salmonella enterica* subsp. *enterica* serovar Muenchen HAZEL-288 |
| HAZEL-289 | PRJNA602741 | SAMN13906192 | JAAHIZ000000000 | *Salmonella enterica* subsp. *enterica* serovar Muenchen HAZEL-289 |
| HAZEL-290 | PRJNA602741 | SAMN13906193 | JAAHTM000000000 | *Salmonella enterica* subsp. *salamae* serovar 1,4,12,27:e,n,x:e,n,x HAZEL-290 |
| HAZEL-291 | PRJNA602741 | SAMN13906194 | JAAHIY000000000 | *Salmonella enterica* subsp. *enterica* serovar Muenchen HAZEL-291 |
| HAZEL-292 | PRJNA602741 | SAMN13906195 | JAAHIX000000000 | *Salmonella enterica* subsp. *enterica* serovar Virchow HAZEL-292 |
| HAZEL-293 | PRJNA602741 | SAMN13906196 | JAAHIW000000000 | *Salmonella enterica* subsp. *enterica* serovar Virchow HAZEL-293 |
| HAZEL-294 | PRJNA602741 | SAMN13906197 | JAAHIV000000000 | *Salmonella enterica* subsp. *enterica* serovar Kentucky HAZEL-294 |
| HAZEL-295 | PRJNA602741 | SAMN13906198 | JAAHIU000000000 | *Salmonella enterica* subsp. *enterica* serovar Kentucky HAZEL-295 |
| HAZEL-296 | PRJNA602741 | SAMN13906199 | JAAHIT000000000 | *Salmonella enterica* subsp. *enterica* serovar Agona HAZEL-296 |
| HAZEL-297 | PRJNA602741 | SAMN13906200 | JAAHSY000000000 | *Salmonella enterica* subsp. *enterica* serovar Agona HAZEL-297 |
| HAZEL-298 | PRJNA602741 | SAMN13906201 | JAAHIS000000000 | *Salmonella enterica* subsp. *enterica* serovar Braenderup HAZEL-298 |
| HAZEL-299 | PRJNA602741 | SAMN13906202 | JAAHIR000000000 | *Salmonella enterica* subsp. *enterica* serovar Braenderup HAZEL-299 |
| HAZEL-300 | PRJNA602741 | SAMN13906203 | JAAHIQ000000000 | *Salmonella enterica* subsp. *enterica* serovar Braenderup HAZEL-300 |
| HAZEL-301 | PRJNA602741 | SAMN13906204 | JAAHIP000000000 | *Salmonella enterica* subsp. *enterica* serovar Braenderup HAZEL-301 |
| HAZEL-302 | PRJNA602741 | SAMN13906205 | JAAHIO000000000 | *Salmonella enterica* subsp. *enterica* serovar Braenderup HAZEL-302 |
| HAZEL-303 | PRJNA602741 | SAMN13906206 | JAAHIN000000000 | *Salmonella enterica* subsp. *enterica* serovar Braenderup HAZEL-303 |
| HAZEL-304 | PRJNA602741 | SAMN13906207 | JAAHIM000000000 | *Salmonella enterica* subsp. *enterica* serovar Braenderup HAZEL-304 |
| HAZEL-305 | PRJNA602741 | SAMN13906208 | JAAHIL000000000 | *Salmonella enterica* subsp. *enterica* serovar Braenderup HAZEL-305 |
| HAZEL-306 | PRJNA602741 | SAMN13906209 | JAAHIK000000000 | *Salmonella enterica* subsp. *enterica* serovar Braenderup HAZEL-306 |
| HAZEL-307 | PRJNA602741 | SAMN13906210 | JAAHIJ000000000 | *Salmonella enterica* subsp. *enterica* serovar Braenderup HAZEL-307 |
| HZ001 | PRJNA602741 | SAMN13906211 | JAAIEQ000000000 | *Salmonella enterica* subsp. *enterica* serovar Typhimurium HZ001 |
| HZ002 | PRJNA602741 | SAMN13906212 | JAAIEP000000000 | *Salmonella enterica* subsp. *enterica* serovar Typhimurium HZ002 |
| HZ003 | PRJNA602741 | SAMN13906213 | JAAIEO000000000 | *Salmonella enterica* subsp. *enterica* serovar Stanleyville HZ003 |
| HZ004 | PRJNA602741 | SAMN13906214 | JAAIEN000000000 | *Salmonella enterica* subsp. *enterica* serovar Enteritidis HZ004 |
| HZ005 | PRJNA602741 | SAMN13906215 | JAAIEM000000000 | *Salmonella enterica* subsp. *enterica* serovar Typhimurium HZ005 |
| HZ006 | PRJNA602741 | SAMN13906216 | JAAIEL000000000 | *Salmonella enterica* subsp. *enterica* serovar Heidelberg HZ006 |
| HZ007 | PRJNA602741 | SAMN13906217 | JAAIEK000000000 | *Salmonella enterica* subsp. *enterica* serovar Aberdeen HZ007 |
| HZ008 | PRJNA602741 | SAMN13906218 | JAAIEJ000000000 | *Salmonella enterica* subsp. *enterica* serovar Enteritidis HZ008 |
| HZ009 | PRJNA602741 | SAMN13906219 | JAAIEI000000000 | *Salmonella enterica* subsp. *enterica* serovar Enteritidis HZ009 |
| HZ010 | PRJNA602741 | SAMN13906220 | JAAIEH000000000 | *Salmonella enterica* subsp. *enterica* serovar Enteritidis HZ010 |
| HZ011 | PRJNA602741 | SAMN13906221 | JAAIFB000000000 | *Salmonella enterica* subsp. *enterica* serovar Enteritidis HZ011 |
| HZ012 | PRJNA602741 | SAMN13906222 | JAAIEG000000000 | *Salmonella enterica* subsp. *enterica* serovar Enteritidis HZ012 |
| HZ013 | PRJNA602741 | SAMN13906223 | JAAIEF000000000 | *Salmonella enterica* subsp. *enterica* serovar Breda HZ013 |
| HZ014 | PRJNA602741 | SAMN13906224 | JAAIGD000000000 | *Salmonella enterica* subsp. *salamae* serovar II 1,13,23:z29:e,n,x HZ014 |
| HZ015 | PRJNA602741 | SAMN13906225 | JAAIEE000000000 | *Salmonella enterica* subsp. *enterica* serovar Typhimurium HZ015 |
| HZ016 | PRJNA602741 | SAMN13906226 | JAAIED000000000 | *Salmonella enterica* subsp. *enterica* serovar Typhimurium HZ016 |
| HZ017 | PRJNA602741 | SAMN13906227 | JAAIEC000000000 | *Salmonella enterica* subsp. *enterica* serovar Typhimurium HZ017 |
| HZ018 | PRJNA602741 | SAMN13906228 | JAAIEB000000000 | *Salmonella enterica* subsp. *enterica* serovar Typhimurium HZ018 |
| HZ019 | PRJNA602741 | SAMN13906229 | JAAIFA000000000 | *Salmonella enterica* subsp. *enterica* serovar Enteritidis HZ019 |
| HZ020 | PRJNA602741 | SAMN13906230 | JAAIEA000000000 | *Salmonella enterica* subsp. *enterica* serovar Newport HZ020 |
| HZ021 | PRJNA602741 | SAMN13906231 | JAAIDZ000000000 | *Salmonella enterica* subsp. *enterica* serovar Enteritidis HZ021 |
| HZ022 | PRJNA602741 | SAMN13906232 | JAAIDY000000000 | *Salmonella enterica* subsp. *enterica* serovar Enteritidis HZ022 |
| HZ023 | PRJNA602741 | SAMN13906233 | JAAIDX000000000 | *Salmonella enterica* subsp. *enterica* serovar Typhimurium HZ023 |
| HZ025 | PRJNA602741 | SAMN13906235 | JAAIDV000000000 | *Salmonella enterica* subsp. *enterica* serovar Typhimurium HZ025 |
| HZ026 | PRJNA602741 | SAMN13906236 | JAAIDU000000000 | *Salmonella enterica* subsp. *enterica* serovar Typhimurium HZ026 |
| HZ027 | PRJNA602741 | SAMN13906237 | JAAIDT000000000 | *Salmonella enterica* subsp. *enterica* serovar Typhimurium HZ027 |
| HZ029 | PRJNA602741 | SAMN13906238 | JAAIDS000000000 | *Salmonella enterica* subsp. *enterica* serovar Enteritidis HZ029 |
| HZ030 | PRJNA602741 | SAMN13906239 | JAAIDR000000000 | *Salmonella enterica* subsp. *enterica* serovar Typhimurium HZ030 |
| HZ031 | PRJNA602741 | SAMN13906240 | JAAIDQ000000000 | *Salmonella enterica* subsp. *enterica* serovar Enteritidis HZ031 |
| HZ032 | PRJNA602741 | SAMN13906241 | JAAIDP000000000 | *Salmonella enterica* subsp. *enterica* serovar Enteritidis HZ032 |
| HZ033 | PRJNA602741 | SAMN13906242 | JAAIDO000000000 | *Salmonella enterica* subsp. *enterica* serovar Virchow HZ033 |
| HZ035 | PRJNA602741 | SAMN13906244 | JAAIDN000000000 | *Salmonella enterica* subsp. *enterica* serovar Typhimurium HZ035 |
| HZ036 | PRJNA602741 | SAMN13906245 | JAAIEZ000000000 | *Salmonella enterica* subsp. *enterica* serovar Typhimurium HZ036 |
| HZ037 | PRJNA602741 | SAMN13906246 | JAAIEY000000000 | *Salmonella enterica* subsp. *enterica* serovar Typhimurium HZ037 |
| HZ038 | PRJNA602741 | SAMN13906247 | JAAIDM000000000 | *Salmonella enterica* subsp. *enterica* serovar Enteritidis HZ038 |
| HZ039 | PRJNA602741 | SAMN13906248 | JAAIEX000000000 | *Salmonella enterica* subsp. *enterica* serovar Typhimurium HZ039 |
| HZ040 | PRJNA602741 | SAMN13906249 | JAAIDL000000000 | *Salmonella enterica* subsp. *enterica* serovar Typhimurium HZ040 |
| HZ041 | PRJNA602741 | SAMN13906250 | JAAIDK000000000 | *Salmonella enterica* subsp. *enterica* serovar Virchow HZ041 |
| HZ042 | PRJNA602741 | SAMN13906251 | JAAIDJ000000000 | *Salmonella enterica* subsp. *enterica* serovar Typhimurium HZ042 |
| HZ043 | PRJNA602741 | SAMN13906252 | JAAIDI000000000 | *Salmonella enterica* subsp. *enterica* serovar Typhimurium HZ043 |
| HZ044 | PRJNA602741 | SAMN13906253 | JAAIDH000000000 | *Salmonella enterica* subsp. *enterica* serovar Typhimurium HZ044 |
| HZ045 | PRJNA602741 | SAMN13906254 | JAAIDG000000000 | *Salmonella enterica* subsp. *enterica* serovar Typhimurium HZ045 |
| HZ046 | PRJNA602741 | SAMN13906255 | JAAIDF000000000 | *Salmonella enterica* subsp. *enterica* serovar Typhimurium HZ046 |
| HZ047 | PRJNA602741 | SAMN13906256 | JAAIDE000000000 | *Salmonella enterica* subsp. *enterica* serovar Typhimurium HZ047 |
| HZ048 | PRJNA602741 | SAMN13906257 | JAAIDD000000000 | *Salmonella enterica* subsp. *enterica* serovar Enteritidis HZ048 |
| HZ050 | PRJNA602741 | SAMN13906258 | JAAIDC000000000 | *Salmonella enterica* subsp. *enterica* serovar Typhimurium HZ050 |
| HZ051 | PRJNA602741 | SAMN13906259 | JAAIDB000000000 | *Salmonella enterica* subsp. *enterica* serovar Typhimurium HZ051 |
| HZ053 | PRJNA602741 | SAMN13906260 | JAAIDA000000000 | *Salmonella enterica* subsp. *enterica* serovar Typhimurium HZ053 |
| HZ055 | PRJNA602741 | SAMN13906262 | JAAICZ000000000 | *Salmonella enterica* subsp. *enterica* serovar Enteritidis HZ055 |
| HZ056 | PRJNA602741 | SAMN13906263 | JAAICY000000000 | *Salmonella enterica* subsp. *enterica* serovar Typhimurium HZ056 |
| HZ057 | PRJNA602741 | SAMN13906264 | JAAICX000000000 | *Salmonella enterica* subsp. *enterica* serovar Enteritidis HZ057 |
| HZ058 | PRJNA602741 | SAMN13906265 | JAAICW000000000 | *Salmonella enterica* subsp. *enterica* serovar Typhimurium HZ058 |
| HZ059 | PRJNA602741 | SAMN13906266 | JAAICV000000000 | *Salmonella enterica* subsp. *enterica* serovar Typhimurium HZ059 |
| HZ061 | PRJNA602741 | SAMN13906268 | JAAICT000000000 | *Salmonella enterica* subsp. *enterica* serovar Typhimurium HZ061 |
| HZ062 | PRJNA602741 | SAMN13906269 | JAAICS000000000 | *Salmonella enterica* subsp. *enterica* serovar Enteritidis HZ062 |
| HZ063 | PRJNA602741 | SAMN13906270 | JAAICR000000000 | *Salmonella enterica* subsp. *enterica* serovar Typhimurium HZ063 |
| HZ064 | PRJNA602741 | SAMN13906271 | JAAICQ000000000 | *Salmonella enterica* subsp. *enterica* serovar Enteritidis HZ064 |
| HZ065 | PRJNA602741 | SAMN13906272 | JAAICP000000000 | *Salmonella enterica* subsp. *enterica* serovar Enteritidis HZ065 |
| HZ066 | PRJNA602741 | SAMN13906273 | JAAICO000000000 | *Salmonella enterica* subsp. *enterica* serovar Enteritidis HZ066 |
| HZ067 | PRJNA602741 | SAMN13906274 | JAAICN000000000 | *Salmonella enterica* subsp. *enterica* serovar Enteritidis HZ067 |
| HZ068 | PRJNA602741 | SAMN13906275 | JAAICM000000000 | *Salmonella enterica* subsp. *enterica* serovar Typhimurium HZ068 |
| HZ069 | PRJNA602741 | SAMN13906276 | JAAICL000000000 | *Salmonella enterica* subsp. *enterica* serovar Enteritidis HZ069 |
| HZ070 | PRJNA602741 | SAMN13906277 | JAAICK000000000 | *Salmonella enterica* subsp. *enterica* serovar Typhimurium HZ070 |
| HZ071 | PRJNA602741 | SAMN13906278 | JAAICJ000000000 | *Salmonella enterica* subsp. *enterica* serovar Enteritidis HZ071 |
| HZ072 | PRJNA602741 | SAMN13906279 | JAAICI000000000 | *Salmonella enterica* subsp. *enterica* serovar Typhimurium HZ072 |
| HZ073 | PRJNA602741 | SAMN13906280 | JAAICH000000000 | *Salmonella enterica* subsp. *enterica* serovar Newport HZ073 |
| HZ074 | PRJNA602741 | SAMN13906281 | JAAICG000000000 | *Salmonella enterica* subsp. *enterica* serovar Typhimurium HZ074 |
| HZ075 | PRJNA602741 | SAMN13906282 | JAAICF000000000 | *Salmonella enterica* subsp. *enterica* serovar Aberdeen HZ075 |
| HZ076 | PRJNA602741 | SAMN13906283 | JAAICE000000000 | *Salmonella enterica* subsp. *enterica* serovar Heidelberg HZ076 |
| HZ077 | PRJNA602741 | SAMN13906284 | JAAICD000000000 | *Salmonella enterica* subsp. *enterica* serovar Typhimurium HZ077 |
| HZ078 | PRJNA602741 | SAMN13906285 | JAAICC000000000 | *Salmonella enterica* subsp. *enterica* serovar Heidelberg HZ078 |
| HZ079 | PRJNA602741 | SAMN13906286 | JAAICB000000000 | *Salmonella enterica* subsp. *enterica* serovar Enteritidis HZ079 |
| HZ080 | PRJNA602741 | SAMN13906287 | JAAICA000000000 | *Salmonella enterica* subsp. *enterica* serovar Enteritidis HZ080 |
| HZ081 | PRJNA602741 | SAMN13906288 | JAAIBZ000000000 | *Salmonella enterica* subsp. *enterica* serovar Heidelberg HZ081 |
| HZ082 | PRJNA602741 | SAMN13906289 | JAAIBY000000000 | *Salmonella enterica* subsp. *enterica* serovar Heidelberg HZ082 |
| HZ083 | PRJNA602741 | SAMN13906290 | JAAIBX000000000 | *Salmonella enterica* subsp. *enterica* serovar Aberdeen HZ083 |
| HZ084 | PRJNA602741 | SAMN13906291 | JAAIBW000000000 | *Salmonella enterica* subsp. *enterica* serovar Fulica HZ084 |
| HZ085 | PRJNA602741 | SAMN13906292 | JAAIBV000000000 | *Salmonella enterica* subsp. *enterica* serovar Enteritidis HZ085 |
| HZ086 | PRJNA602741 | SAMN13906293 | JAAIBU000000000 | *Salmonella enterica* subsp. *enterica* serovar Heidelberg HZ086 |
| HZ087 | PRJNA602741 | SAMN13906294 | JAAIBT000000000 | *Salmonella enterica* subsp. *enterica* serovar Indiana HZ087 |
| HZ088 | PRJNA602741 | SAMN13906295 | JAAIBS000000000 | *Salmonella enterica* subsp. *enterica* serovar Saintpaul HZ088 |
| HZ089 | PRJNA602741 | SAMN13906296 | JAAIBR000000000 | *Salmonella enterica* subsp. *enterica* serovar Typhimurium HZ089 |
| HZ090 | PRJNA602741 | SAMN13906297 | JAAIBQ000000000 | *Salmonella enterica* subsp. *enterica* serovar Enteritidis HZ090 |
| HZ091 | PRJNA602741 | SAMN13906298 | JAAIBP000000000 | *Salmonella enterica* subsp. *enterica* serovar Bovismorbificans HZ091 |
| HZ092 | PRJNA602741 | SAMN13906299 | JAAIBO000000000 | *Salmonella enterica* subsp. *enterica* serovar Enteritidis HZ092 |
| HZ093 | PRJNA602741 | SAMN13906300 | JAAIBN000000000 | *Salmonella enterica* subsp. *enterica* serovar Bovismorbificans HZ093 |
| HZ094 | PRJNA602741 | SAMN13906301 | JAAIBM000000000 | *Salmonella enterica* subsp. *salamae* serovar 42:r:- HZ094 |
| HZ095 | PRJNA602741 | SAMN13906302 | JAAIBL000000000 | *Salmonella enterica* subsp. *enterica* serovar Infantis HZ095 |
| HZ096 | PRJNA602741 | SAMN13906303 | JAAIBK000000000 | *Salmonella enterica* subsp. *enterica* serovar Typhimurium HZ096 |
| HZ097 | PRJNA602741 | SAMN13906304 | JAAIBJ000000000 | *Salmonella enterica* subsp. *enterica* serovar Typhimurium HZ097 |
| HZ098 | PRJNA602741 | SAMN13906305 | JAANPY000000000 | *Salmonella enterica* subsp. *enterica* HZ098 |
| HZ100 | PRJNA602741 | SAMN13906306 | JAAIBI000000000 | *Salmonella enterica* subsp. *enterica* serovar Enteritidis HZ100 |
| HZ101 | PRJNA602741 | SAMN13906307 | JAAIBH000000000 | *Salmonella enterica* subsp. *enterica* serovar Typhimurium HZ101 |
| HZ102 | PRJNA602741 | SAMN13906308 | JAAIEW000000000 | *Salmonella enterica* subsp. *enterica* serovar Typhimurium HZ102 |
| HZ103 | PRJNA602741 | SAMN13906309 | JAAIBG000000000 | *Salmonella enterica* subsp. *enterica* serovar Kenya HZ103 |
| HZ104 | PRJNA602741 | SAMN13906310 | JAAIBF000000000 | *Salmonella enterica* subsp. *enterica* serovar Typhimurium HZ104 |
| HZ105 | PRJNA602741 | SAMN13906311 | JAAIBE000000000 | *Salmonella enterica* subsp. *enterica* serovar Uganda HZ105 |
| HZ106 | PRJNA602741 | SAMN13906312 | JAAIBD000000000 | *Salmonella enterica* subsp. *enterica* serovar Aberdeen HZ106 |
| HZ107 | PRJNA602741 | SAMN13906313 | JAAIGC000000000 | *Salmonella enterica* subsp. *salamae* serovar II 1,13,23:z29:e,n,x HZ107 |
| HZ108 | PRJNA602741 | SAMN13906314 | JAAIBC000000000 | *Salmonella enterica* subsp. *enterica* serovar Heidelberg HZ108 |
| HZ109 | PRJNA602741 | SAMN13906315 | JAAIBB000000000 | *Salmonella enterica* subsp. *enterica* serovar Uganda HZ109 |
| HZ110 | PRJNA602741 | SAMN13906316 | JAAIBA000000000 | *Salmonella enterica* subsp. *enterica* serovar Kenya HZ110 |
| HZ111 | PRJNA602741 | SAMN13906317 | JAAIAZ000000000 | *Salmonella enterica* subsp. *enterica* serovar Uganda HZ111 |
| HZ112 | PRJNA602741 | SAMN13906318 | JAAIAY000000000 | *Salmonella enterica* subsp. *enterica* serovar Breda HZ112 |
| HZ113 | PRJNA602741 | SAMN13906319 | JAAIAX000000000 | *Salmonella enterica* subsp. *enterica* serovar Enteritidis HZ113 |
| HZ114 | PRJNA602741 | SAMN13906320 | JAAIAW000000000 | *Salmonella enterica* subsp. *enterica* serovar Typhimurium HZ114 |
| HZ115 | PRJNA602741 | SAMN13906321 | JAAIAV000000000 | *Salmonella enterica* subsp. *enterica* serovar Typhimurium HZ115 |
| HZ116 | PRJNA602741 | SAMN13906322 | JAAIAU000000000 | *Salmonella enterica* subsp. *enterica* serovar Enteritidis HZ116 |
| HZ117 | PRJNA602741 | SAMN13906323 | JAAIAT000000000 | *Salmonella enterica* subsp. *enterica* serovar Typhimurium HZ117 |
| HZ118 | PRJNA602741 | SAMN13906324 | JAAIAS000000000 | *Salmonella enterica* subsp. *enterica* serovar Newport HZ118 |
| HZ119 | PRJNA602741 | SAMN13906325 | JAAIAR000000000 | *Salmonella enterica* subsp. *enterica* serovar Typhimurium HZ119 |
| HZ120 | PRJNA602741 | SAMN13906326 | JAAIAQ000000000 | *Salmonella enterica* subsp. *enterica* serovar Typhimurium HZ120 |
| HZ121 | PRJNA602741 | SAMN13906327 | JAAIAP000000000 | *Salmonella enterica* subsp. *enterica* serovar Enteritidis HZ121 |
| HZ122 | PRJNA602741 | SAMN13906328 | JAAIAO000000000 | *Salmonella enterica* subsp. *enterica* serovar Enteritidis HZ122 |
| HZ123 | PRJNA602741 | SAMN13906329 | JAAIAN000000000 | *Salmonella enterica* subsp. *enterica* serovar Enteritidis HZ123 |
| HZ124 | PRJNA602741 | SAMN13906330 | JAAIAM000000000 | *Salmonella enterica* subsp. *enterica* serovar Typhimurium HZ124 |
| HZ125 | PRJNA602741 | SAMN13906331 | JAAIAL000000000 | *Salmonella enterica* subsp. *enterica* serovar Typhimurium HZ125 |
| HZ126 | PRJNA602741 | SAMN13906332 | JAAIAK000000000 | *Salmonella enterica* subsp. *enterica* serovar Berkeley HZ126 |
| HZ127 | PRJNA602741 | SAMN13906333 | JAAIAJ000000000 | *Salmonella enterica* subsp. *enterica* serovar Heidelberg HZ127 |
| HZ128 | PRJNA602741 | SAMN13906334 | JAAIAI000000000 | *Salmonella enterica* subsp. *enterica* serovar Typhimurium HZ128 |
| HZ129 | PRJNA602741 | SAMN13906335 | JAAIAH000000000 | *Salmonella enterica* subsp. *enterica* serovar Typhimurium HZ129 |
| HZ130 | PRJNA602741 | SAMN13906336 | JAAIAG000000000 | *Salmonella enterica* subsp. *enterica* serovar Enteritidis HZ130 |
| HZ131 | PRJNA602741 | SAMN13906337 | JAAIAF000000000 | *Salmonella enterica* subsp. *enterica* serovar Heidelberg HZ131 |
| HZ132 | PRJNA602741 | SAMN13906338 | JAAIAE000000000 | *Salmonella enterica* subsp. *enterica* serovar Butantan HZ132 |
| HZ133 | PRJNA602741 | SAMN13906339 | JAAIAD000000000 | *Salmonella enterica* subsp. *enterica* serovar Typhimurium HZ133 |
| HZ134 | PRJNA602741 | SAMN13906340 | JAAIAC000000000 | *Salmonella enterica* subsp. *enterica* serovar Typhimurium HZ134 |
| HZ135 | PRJNA602741 | SAMN13906341 | JAAIAB000000000 | *Salmonella enterica* subsp. *enterica* serovar Enteritidis HZ135 |
| HZ136 | PRJNA602741 | SAMN13906342 | JAAIEV000000000 | *Salmonella enterica* subsp. *enterica* serovar Typhimurium HZ136 |
| HZ137 | PRJNA602741 | SAMN13906343 | JAAIAA000000000 | *Salmonella enterica* subsp. *enterica* serovar Uganda HZ137 |
| HZ138 | PRJNA602741 | SAMN13906344 | JAAIEU000000000 | *Salmonella enterica* subsp. *enterica* serovar Enteritidis HZ138 |
| HZ139 | PRJNA602741 | SAMN13906345 | JAAHZZ000000000 | *Salmonella enterica* subsp. *enterica* serovar Typhimurium HZ139 |
| HZ140 | PRJNA602741 | SAMN13906346 | JAAHZY000000000 | *Salmonella enterica* subsp. *enterica* serovar Typhimurium HZ140 |
| HZ141 | PRJNA602741 | SAMN13906347 | JAAHZX000000000 | *Salmonella enterica* subsp. *enterica* serovar Typhimurium HZ141 |
| HZ142 | PRJNA602741 | SAMN13906348 | JAAHZW000000000 | *Salmonella enterica* subsp. *enterica* serovar Oskarshamn HZ142 |
| HZ143 | PRJNA602741 | SAMN13906349 | JAAHZV000000000 | *Salmonella enterica* subsp. *enterica* serovar Typhimurium HZ143 |
| HZ144 | PRJNA602741 | SAMN13906350 | JAAHZU000000000 | *Salmonella enterica* subsp. *enterica* serovar Uganda HZ144 |
| HZ145 | PRJNA602741 | SAMN13906351 | JAAHZT000000000 | *Salmonella enterica* subsp. *enterica* serovar Aberdeen HZ145 |
| HZ146 | PRJNA602741 | SAMN13906352 | JAAHZS000000000 | *Salmonella enterica* subsp. *enterica* serovar Breda HZ146 |
| HZ147 | PRJNA602741 | SAMN13906353 | JAAHZR000000000 | *Salmonella enterica* subsp. *enterica* serovar Enteritidis HZ147 |
| HZ148 | PRJNA602741 | SAMN13906354 | JAAHZQ000000000 | *Salmonella enterica* subsp. *enterica* serovar Enteritidis HZ148 |
| HZ149 | PRJNA602741 | SAMN13906355 | JAAHZP000000000 | *Salmonella enterica* subsp. *enterica* serovar Enteritidis HZ149 |
| HZ150 | PRJNA602741 | SAMN13906356 | JAAHZO000000000 | *Salmonella enterica* subsp. *enterica* serovar Typhimurium HZ150 |
| HZ151 | PRJNA602741 | SAMN13906357 | JAAHZN000000000 | *Salmonella enterica* subsp. *enterica* serovar Typhimurium HZ151 |
| HZ152 | PRJNA602741 | SAMN13906358 | JAAHZM000000000 | *Salmonella enterica* subsp. *enterica* serovar Enteritidis HZ152 |
| HZ153 | PRJNA602741 | SAMN13906359 | JAAHZL000000000 | *Salmonella enterica* subsp. *enterica* serovar Enteritidis HZ153 |
| HZ154 | PRJNA602741 | SAMN13906360 | JAAHZK000000000 | *Salmonella enterica* subsp. *enterica* serovar Enteritidis HZ154 |
| HZ155 | PRJNA602741 | SAMN13906361 | JAAHZJ000000000 | *Salmonella enterica* subsp. *enterica* serovar Enteritidis HZ155 |
| HZ156 | PRJNA602741 | SAMN13906362 | JAAHZI000000000 | *Salmonella enterica* subsp. *enterica* serovar Heidelberg HZ156 |
| HZ157 | PRJNA602741 | SAMN13906363 | JAAHZH000000000 | *Salmonella enterica* subsp. *enterica* serovar Enteritidis HZ157 |
| HZ160 | PRJNA602741 | SAMN13906365 | JAAHZG000000000 | *Salmonella enterica* subsp. *enterica* serovar Typhimurium HZ160 |
| HZ161 | PRJNA602741 | SAMN13906366 | JAAHZF000000000 | *Salmonella enterica* subsp. *enterica* serovar Typhimurium HZ161 |
| HZ162 | PRJNA602741 | SAMN13906367 | JAAHZE000000000 | *Salmonella enterica* subsp. *enterica* serovar Typhimurium HZ162 |
| HZ164 | PRJNA602741 | SAMN13906369 | JAAHZD000000000 | *Salmonella enterica* subsp. *enterica* serovar Enteritidis HZ164 |
| HZ165 | PRJNA602741 | SAMN13906370 | JAAIET000000000 | *Salmonella enterica* subsp. *enterica* serovar Muenchen HZ165 |
| HZ166 | PRJNA602741 | SAMN13906371 | JAAHZC000000000 | *Salmonella enterica* subsp. *enterica* serovar Newport HZ166 |
| HZ167 | PRJNA602741 | SAMN13906372 | JAAHZB000000000 | *Salmonella enterica* subsp. *enterica* serovar Typhimurium HZ167 |
| HZ168 | PRJNA602741 | SAMN13906373 | JAAHZA000000000 | *Salmonella enterica* subsp. *enterica* serovar Typhimurium HZ168 |
| HZ169 | PRJNA602741 | SAMN13906374 | JAAHYZ000000000 | *Salmonella enterica* subsp. *enterica* serovar Enteritidis HZ169 |
| HZ170 | PRJNA602741 | SAMN13906375 | JAAHYY000000000 | *Salmonella enterica* subsp. *enterica* serovar Enteritidis HZ170 |
| HZ172 | PRJNA602741 | SAMN13906377 | JAAHYW000000000 | *Salmonella enterica* subsp. *enterica* serovar Enteritidis HZ172 |
| HZ173 | PRJNA602741 | SAMN13906378 | JAAHYV000000000 | *Salmonella enterica* subsp. *enterica* serovar Typhimurium HZ173 |
| HZ174 | PRJNA602741 | SAMN13906379 | JAAHYU000000000 | *Salmonella enterica* subsp. *enterica* serovar Typhimurium HZ174 |
| HZ175 | PRJNA602741 | SAMN13906380 | JAAHYT000000000 | *Salmonella enterica* subsp. *enterica* serovar Typhimurium HZ175 |
| HZ176 | PRJNA602741 | SAMN13906381 | JAAHYS000000000 | *Salmonella enterica* subsp. *enterica* serovar Enteritidis HZ176 |
| HZ177 | PRJNA602741 | SAMN13906382 | JAAHYR000000000 | *Salmonella enterica* subsp. *enterica* serovar Enteritidis HZ177 |
| HZ178 | PRJNA602741 | SAMN13906383 | JAAHYQ000000000 | *Salmonella enterica* subsp. *enterica* serovar Enteritidis HZ178 |
| HZ179 | PRJNA602741 | SAMN13906384 | JAAHYP000000000 | *Salmonella enterica* subsp. *enterica* serovar Typhimurium HZ179 |
| HZ180 | PRJNA602741 | SAMN13906385 | JAAHYO000000000 | *Salmonella enterica* subsp. *enterica* serovar Enteritidis HZ180 |
| HZ182 | PRJNA602741 | SAMN13906386 | JAAHYN000000000 | *Salmonella enterica* subsp. *enterica* serovar Typhimurium HZ182 |
| HZ183 | PRJNA602741 | SAMN13906387 | JAAHYM000000000 | *Salmonella enterica* subsp. *enterica* serovar Typhimurium HZ183 |
| HZ184 | PRJNA602741 | SAMN13906388 | JAAHYL000000000 | *Salmonella enterica* subsp. *enterica* serovar Typhimurium HZ184 |
| HZ185 | PRJNA602741 | SAMN13906389 | JAAHYK000000000 | *Salmonella enterica* subsp. *enterica* serovar Typhimurium HZ185 |
| HZ200 | PRJNA602741 | SAMN13906390 | JAANPX000000000 | *Salmonella enterica* subsp. *enterica* HZ200 |
| HZ201 | PRJNA602741 | SAMN13906391 | JAAHYJ000000000 | *Salmonella enterica* subsp. *enterica* serovar Kentucky HZ201 |
| HZ202 | PRJNA602741 | SAMN13906392 | JAAHYI000000000 | *Salmonella enterica* subsp. *enterica* serovar Kentucky HZ202 |
| HZ212 | PRJNA602741 | SAMN13906393 | JAAHYH000000000 | *Salmonella enterica* subsp. *enterica* serovar Enteritidis HZ212 |
| HZ213 | PRJNA602741 | SAMN13906394 | JAAHYG000000000 | *Salmonella enterica* subsp. *enterica* serovar Enteritidis HZ213 |
| HZ214 | PRJNA602741 | SAMN13906395 | JAAHYF000000000 | *Salmonella enterica* subsp. *enterica* serovar Durban HZ214 |
| HZ215 | PRJNA602741 | SAMN13906396 | JAAHYE000000000 | *Salmonella enterica* subsp. *enterica* serovar Durban HZ215 |
| HZ216 | PRJNA602741 | SAMN13906397 | JAAHYD000000000 | *Salmonella enterica* subsp. *enterica* serovar Durban HZ216 |
| HZ217 | PRJNA602741 | SAMN13906398 | JAAHYC000000000 | *Salmonella enterica* subsp. *enterica* serovar Durban HZ217 |
| HZ218 | PRJNA602741 | SAMN13906399 | JAAHYB000000000 | *Salmonella enterica* subsp. *enterica* serovar Newport HZ218 |
| HZ219 | PRJNA602741 | SAMN13906400 | JAAHYA000000000 | *Salmonella enterica* subsp. *enterica* serovar Newport HZ219 |
| HZ222 | PRJNA602741 | SAMN13906401 | JAAHXZ000000000 | *Salmonella enterica* subsp. *enterica* serovar Typhimurium HZ222 |
| HZ223 | PRJNA602741 | SAMN13906402 | JAAHXY000000000 | *Salmonella enterica* subsp. *enterica* serovar Typhimurium HZ223 |
| HZ227 | PRJNA602741 | SAMN13906403 | JAAHXX000000000 | *Salmonella enterica* subsp. *enterica* serovar Cerro HZ227 |
| HZ228 | PRJNA602741 | SAMN13906404 | JAAHXW000000000 | *Salmonella enterica* subsp. *enterica* serovar Leoben HZ228 |
| HZ229 | PRJNA602741 | SAMN13906405 | JAAHXV000000000 | *Salmonella enterica* subsp. *enterica* serovar Kentucky HZ229 |
| HZ230 | PRJNA602741 | SAMN13906406 | JAAHXU000000000 | *Salmonella enterica* subsp. *enterica* serovar Kentucky HZ230 |
| HZ231 | PRJNA602741 | SAMN13906407 | JAAHXT000000000 | *Salmonella enterica* subsp. *salamae* serovar 47:b:e,n,x,z15 HZ231 |
| HZ232 | PRJNA602741 | SAMN13906408 | JAAHXS000000000 | *Salmonella enterica* subsp. *salamae* serovar 47:b:e,n,x,z15 HZ232 |
| HZ233 | PRJNA602741 | SAMN13906409 | JAAHXR000000000 | *Salmonella enterica* subsp. *enterica* serovar Typhimurium HZ233 |
| HZ234 | PRJNA602741 | SAMN13906410 | JAAHXQ000000000 | *Salmonella enterica* subsp. *enterica* serovar Typhimurium HZ234 |
| HZ235 | PRJNA602741 | SAMN13906411 | JAAHXP000000000 | *Salmonella enterica* subsp. *enterica* serovar Breda HZ235 |
| HZ236 | PRJNA602741 | SAMN13906412 | JAAHXO000000000 | *Salmonella enterica* subsp. *enterica* serovar Breda HZ236 |
| HZ237 | PRJNA602741 | SAMN13906413 | JAAHXN000000000 | *Salmonella enterica* subsp. *enterica* serovar Orion HZ237 |
| HZ238 | PRJNA602741 | SAMN13906414 | JAAHXM000000000 | *Salmonella enterica* subsp. *enterica* serovar Orion HZ238 |
| HZ239 | PRJNA602741 | SAMN13906415 | JAAHXL000000000 | *Salmonella enterica* subsp. *enterica* serovar Virchow HZ239 |
| HZ240 | PRJNA602741 | SAMN13906416 | JAAHXK000000000 | *Salmonella enterica* subsp. *enterica* serovar Aberdeen HZ240 |
| HZ241 | PRJNA602741 | SAMN13906417 | JAAHXJ000000000 | *Salmonella enterica* subsp. *enterica* serovar Aberdeen HZ241 |
| HZ242 | PRJNA602741 | SAMN13906418 | JAAHXI000000000 | *Salmonella enterica* subsp. *enterica* serovar Aberdeen HZ242 |
| HZ243 | PRJNA602741 | SAMN13906419 | JAAIGB000000000 | *Salmonella enterica* subsp. *salamae* serovar II 1,13,23:z29:e,n,x HZ243 |
| HZ244 | PRJNA602741 | SAMN13906420 | JAAHXH000000000 | *Salmonella enterica* subsp. *enterica* serovar Colindale HZ244 |
| HZ245 | PRJNA602741 | SAMN13906421 | JAAIGA000000000 | *Salmonella enterica* subsp. *salamae* serovar II 1,4,12,27:e,n,x:e,n,x HZ245 |
| HZ246 | PRJNA602741 | SAMN13906422 | JAAIFZ000000000 | *Salmonella enterica* subsp. *salamae* serovar II 1,4,12,27:e,n,x:e,n,x HZ246 |
| HZ247 | PRJNA602741 | SAMN13906423 | JAAIFY000000000 | *Salmonella enterica* subsp. *salamae* serovar II 1,4,12,27:e,n,x:e,n,x HZ247 |
| HZ248 | PRJNA602741 | SAMN13906424 | JAAHXG000000000 | *Salmonella enterica* subsp. *enterica* serovar Braenderup HZ248 |
| HZ249 | PRJNA602741 | SAMN13906425 | JAAIES000000000 | *Salmonella enterica* subsp. *enterica* serovar Braenderup HZ249 |
| HZ250 | PRJNA602741 | SAMN13906426 | JAAHXF000000000 | *Salmonella enterica* subsp. *enterica* serovar Agona HZ250 |
| HZ251 | PRJNA602741 | SAMN13906427 | JAAHXE000000000 | *Salmonella enterica* subsp. *enterica* serovar Typhimurium HZ251 |
| HZ252 | PRJNA602741 | SAMN13906428 | JAAHXD000000000 | *Salmonella enterica* subsp. *salamae* serovar 42:r:- HZ252 |
| HZ253 | PRJNA602741 | SAMN13906429 | JAAHXC000000000 | *Salmonella enterica* subsp. *salamae* serovar 42:r:- HZ253 |
| HZ254 | PRJNA602741 | SAMN13906430 | JAAHXB000000000 | *Salmonella enterica* subsp. *salamae* serovar 42:r:- HZ254 |
| HZ255 | PRJNA602741 | SAMN13906431 | JAAHXA000000000 | *Salmonella enterica* subsp. *salamae* serovar 42:r:- HZ255 |
| HZ256 | PRJNA602741 | SAMN13906432 | JAAHWZ000000000 | *Salmonella enterica* subsp. *enterica* serovar Sundsvall HZ256 |
| HZ257 | PRJNA602741 | SAMN13906433 | JAAIFX000000000 | *Salmonella enterica* subsp. *salamae* serovar II 1,4,12,27:e,n,x:e,n,x HZ257 |
| HZ258 | PRJNA602741 | SAMN13906434 | JAAIFW000000000 | *Salmonella enterica* subsp. *salamae* serovar II 1,4,12,27:e,n,x:e,n,x HZ258 |
| HZ259 | PRJNA602741 | SAMN13906435 | JAAIFV000000000 | *Salmonella enterica* subsp. *salamae* serovar II 1,4,12,27:e,n,x:e,n,x HZ259 |
| HZ260 | PRJNA602741 | SAMN13906436 | JAAHWY000000000 | *Salmonella enterica* subsp. *enterica* serovar Enteritidis HZ260 |
| HZ261 | PRJNA602741 | SAMN13906437 | JAAHWX000000000 | *Salmonella enterica* subsp. *enterica* serovar Enteritidis HZ261 |
| HZ262 | PRJNA602741 | SAMN13906438 | JAAHWW000000000 | *Salmonella enterica* subsp. *enterica* serovar Muenchen HZ262 |
| HZ263 | PRJNA602741 | SAMN13906439 | JAAHWV000000000 | *Salmonella enterica* subsp. *enterica* serovar Agona HZ263 |
| HZ264 | PRJNA602741 | SAMN13906440 | JAAHWU000000000 | *Salmonella enterica* subsp. *enterica* serovar Saintpaul HZ264 |
| HZ265 | PRJNA602741 | SAMN13906441 | JAAHWT000000000 | *Salmonella enterica* subsp. *enterica* serovar Saintpaul HZ265 |
| HZ266 | PRJNA602741 | SAMN13906442 | JAAHWS000000000 | *Salmonella enterica* subsp. *enterica* serovar Kiambu HZ266 |
| HZ267 | PRJNA602741 | SAMN13906443 | JAAHWR000000000 | *Salmonella enterica* subsp. *enterica* serovar Kiambu HZ267 |
| HZ268 | PRJNA602741 | SAMN13906444 | JAAHWQ000000000 | *Salmonella enterica* subsp. *enterica* serovar Eastbourne HZ268 |
| HZ269 | PRJNA602741 | SAMN13906445 | JAAHWP000000000 | *Salmonella enterica* subsp. *enterica* serovar Durban HZ269 |
| HZ270 | PRJNA602741 | SAMN13906446 | JAAHWO000000000 | *Salmonella enterica* subsp. *enterica* serovar Durban HZ270 |
| HZ271 | PRJNA602741 | SAMN13906447 | JAAHWN000000000 | *Salmonella enterica* subsp. *enterica* serovar Durban HZ271 |
| HZ272 | PRJNA602741 | SAMN13906448 | JAAHWM000000000 | *Salmonella enterica* subsp. *enterica* serovar Durban HZ272 |
| HZ273 | PRJNA602741 | SAMN13906449 | JAAHWL000000000 | *Salmonella enterica* subsp. *enterica* serovar Typhimurium HZ273 |
| HZ274 | PRJNA602741 | SAMN13906450 | JAAHWK000000000 | *Salmonella enterica* subsp. *enterica* serovar Virchow HZ274 |
| HZ275 | PRJNA602741 | SAMN13906451 | JAAHWJ000000000 | *Salmonella enterica* subsp. *enterica* serovar Durban HZ275 |
| HZ276 | PRJNA602741 | SAMN13906452 | JAAHWI000000000 | *Salmonella enterica* subsp. *enterica* serovar Orion HZ276 |
| HZ277 | PRJNA602741 | SAMN13906453 | JAAHWH000000000 | *Salmonella enterica* subsp. *enterica* serovar Orion HZ277 |
| HZ278 | PRJNA602741 | SAMN13906454 | JAAIFU000000000 | *Salmonella enterica* subsp. *salamae* serovar II 48:b:- HZ278 |
| HZ279 | PRJNA602741 | SAMN13906455 | JAAIFT000000000 | *Salmonella enterica* subsp. *salamae* serovar II 48:b:- HZ279 |
| HZ280 | PRJNA602741 | SAMN13906456 | JAAIER000000000 | *Salmonella enterica* subsp. *enterica* serovar Typhimurium HZ280 |
| ZLM0214b | PRJNA602741 | SAMN13906457 | JAAHWG000000000 | *Salmonella enterica* subsp. *enterica* serovar Give ZLM0214b |
