# Supplementary material for: Investigating the Meat Pathway as a Source of Human Nontyphoidal Salmonella Bloodstream Infections and Diarrhea in East Africa
Source: Clin Infect Dis. 2020 Aug 10;73(7):e1570–8. doi: 10.1093/cid/ciaa1153 (PMC8492120; doi:10.1093/cid/ciaa1153)
Supplement: ciaa1153_suppl_Supplementary_Table_S2 [file ciaa1153_suppl_supplementary_table_s2.docx]

**Supplementary Table 2. *Salmonella* serovars and multi-locus sequence types by sample source, East Africa, 2007-17**

|  | **Sample source** | | | | | | | | | |
| --- | --- | --- | --- | --- | --- | --- | --- | --- | --- | --- |
| ***Salmonella* serovar** | ***Salmonella* multi-locus sequence type*** | **n** | **Poultry farm environment** | **Poultry cloaca** | **Slaughter and butcher environment** | **Cattle and goat intestinal samples** | **Cattle and goat carcass** | **Cattle and goat meat** | **Human feces** | **Human blood** |
| **Typhimurium** |  | **91** | **1** | **4** | **6** | **3** | **1** | **3** | **26** | **47** |
|  | **19** | **24** | **1** | **4** | **1** | **3** | **1** | **3** | **9** | **2** |
|  | **313** | **62** |  |  |  |  |  |  | **17** | **45** |
|  | **UNK1** | **5** |  |  | **5** |  |  |  |  |  |
| **Enteritidis** | **11** | **78** | **6** | **9** |  | **4** | **2** | **4** | **21** | **32** |
| **Orion** |  | **34** |  |  | **7** | **2** | **2** | **23** |  |  |
|  | **639** | **33** |  |  | **7** | **2** | **2** | **22** |  |  |
|  | **UNK12** | **1** |  |  |  |  |  | **1** |  |  |
| **II 42:r:-** | **1208** | **28** |  |  | **5** |  | **4** | **18** | **1** |  |
| **Kentucky** |  | **19** | **2** | **7** | **1** |  | **1** | **8** |  |  |
|  | **198** | **13** | **2** | **7** |  |  |  | **4** |  |  |
|  | **314** | **6** |  |  | **1** |  | **1** | **4** |  |  |
| **Newport** |  | **18** | **5** | **6** | **1** |  |  | **2** | **3** | **1** |
|  | **31** | **1** |  |  |  |  |  |  |  | **1** |
|  | **46** | **3** |  |  |  |  |  |  | **3** |  |
|  | **166** | **14** | **5** | **6** | **1** |  |  | **2** |  |  |
| **Saintpaul** | **27** | **18** | **2** |  | **2** |  | **2** | **11** | **1** |  |
| **Virchow** | **16** | **15** | **3** | **3** |  |  |  | **7** | **1** | **1** |
| **Braenderup** | **22** | **14** |  |  | **14** |  |  |  |  |  |
| **Heidelberg** | **15** | **14** |  |  |  |  |  | **4** | **9** | **1** |
| **Karamoja** | **912** | **14** |  |  | **7** | **2** |  | **5** |  |  |
| **Durban** | **2533** | **13** | **4** | **4** |  | **3** |  | **2** |  |  |
| **II 1,4,12,27:e,n,x:e,n,x** | **UNK11** | **11** |  |  | **11** |  |  |  |  |  |
| **Jangwani** |  | **11** |  |  | **3** |  |  | **8** |  |  |
|  | **UNK3** | **6** |  |  |  |  |  | **6** |  |  |
|  | **UNK5** | **5** |  |  | **3** |  |  | **2** |  |  |
| **Aberdeen** |  | **10** |  |  | **5** |  |  |  | **5** |  |
|  | **3610** | **5** |  |  | **5** |  |  |  |  |  |
|  | **UNK20** | **5** |  |  |  |  |  |  | **5** |  |
| **Agona** | **13** | **9** | **3** |  | **4** |  | **1** | **1** |  |  |
| **II 1,13,23:z29:e,n,x** | **1188** | **8** |  |  | **6** |  |  |  | **2** |  |
| **Kibusi** | **UNK7** | **8** |  |  |  |  |  | **8** |  |  |
| **Breda** | **582** | **7** |  |  | **2** |  | **2** |  | **3** |  |
| **Eastbourne** | **414** | **7** | **1** |  | **2** |  | **3** | **1** |  |  |
| **II 1,13,23:z:1,5** | **1018** | **7** |  |  | **2** |  |  | **5** |  |  |
| **Kiambu** | **309** | **6** | **2** |  | **1** | **1** |  | **2** |  |  |
| **Kisarawe** | **UNK2** | **7** |  |  | **3** |  |  | **4** |  |  |
| **Muenchen** | **82** | **7** | **4** | **2** |  |  |  |  | **1** |  |
| **Indiana** | **2040** | **5** |  |  |  |  |  | **4** | **1** |  |
| **Uganda** | **684** | **5** |  |  |  |  |  |  | **5** |  |
| **Give** | **516** | **4** |  |  |  |  |  | **4** |  |  |
| **Hadar** | **473** | **4** |  |  |  | **2** |  | **2** |  |  |
| **II 1,9,12,46,27:l,w:e,n,x** | **103** | **4** |  |  | **4** |  |  |  |  |  |
| **Leoben** | **1405** | **4** |  |  | **2** | **1** |  | **1** |  |  |
| **Livingstone** | **2587** | **4** |  |  |  |  |  | **4** |  |  |
| **Nyborg\|Sanktmarx** | **UNK4** | **4** |  |  |  |  | **2** | **2** |  |  |
| **Poona** |  | **4** |  |  | **1** |  |  | **3** |  |  |
|  | **714** | **1** |  |  |  |  |  | **1** |  |  |
|  | **2566** | **3** |  |  | **1** |  |  | **2** |  |  |
| **Bahrenfeld** | **1458** | **2** |  |  |  |  |  | **2** |  |  |
| **Bovismorbificans** | **142** | **2** |  |  |  |  |  |  | **2** |  |
| **Epinay** | **UNK9** | **2** |  |  | **2** |  |  |  |  |  |
| **Hvittingfoss** | **446** | **2** |  |  |  |  |  | **2** |  |  |
| **I 9,46:[g,t]:-** | **UNK6** | **2** |  |  |  |  |  | **2** |  |  |
| **II 28:g,m,t:e,n,x\|II 28:m,t:[e,n,x]** | **UNK8** | **2** |  |  | **2** |  |  |  |  |  |
| **II 47:b:e,n,x,z15** | **UNK19** | **2** |  | **2** |  |  |  |  |  |  |
| **II 48:b:-** | **UNK18** | **2** |  |  |  |  |  | **2** |  |  |
| **II 6,7:g,[m],s,t:[z42]\|II 6,7:m,t:-** | **3510** | **2** |  | **2** |  |  |  |  |  |  |
| **Jedburgh\|Llandoff** | **UNK10** | **2** |  |  |  |  |  | **2** |  |  |
| **Johannesburg** | **512** | **2** |  |  |  | **2** |  |  |  |  |
| **Kenya** | **991** | **2** |  |  |  |  |  |  | **2** |  |
| **Onderstepoort** | **1458** | **2** |  |  |  |  |  | **2** |  |  |
| **Senftenberg** | **14** | **2** |  |  | **2** |  |  |  |  |  |
| **Umbilo** | **2014** | **2** |  |  |  |  |  | **2** |  |  |
| **Yarrabah** | **536** | **2** |  |  | **2** |  |  |  |  |  |
| **Annedal\|Kibusi\|Rubislaw\|Yellowknife** | **UNK7** | **1** |  |  |  |  |  | **1** |  |  |
| **Baildon\|Lomalinda** | **988** | **1** |  |  |  | **1** |  |  |  |  |
| **Berkeley** | **3242** | **1** |  |  |  |  |  |  | **1** |  |
| **Bullbay\|Potsdam** | **UNK15** | **1** |  |  |  |  |  |  | **1** |  |
| **Butantan** | **600** | **1** |  |  |  |  |  |  | **1** |  |
| **Cerro** | **UNK13** | **1** |  |  |  | **1** |  |  |  |  |
| **Chandans** | **465** | **1** |  |  |  | **1** |  |  |  |  |
| **Colindale** | **584** | **1** | **1** |  |  |  |  |  |  |  |
| **Fulica** | **UNK14** | **1** |  |  |  |  |  |  | **1** |  |
| **Infantis** | **603** | **1** |  |  |  |  |  |  | **1** |  |
| **Kingabwa** | **546** | **1** |  |  |  |  |  | **1** |  |  |
| **Oskarshamn** | **UNK16** | **1** |  |  |  |  |  |  | **1** |  |
| **Richmond** | **909** | **1** |  |  |  |  |  | **1** |  |  |
| **Stanleyville** | **UNK17** | **1** |  |  |  |  |  |  | **1** |  |
| **Sundsvall** | **488** | **1** |  |  |  | **1** |  |  |  |  |
| **Total** |  | **539** | **34** | **39** | **97** | **24** | **20** | **153** | **90** | **82** |

UNK = unknown

*Unknown MLSTs were assigned a grouping UNK 1-20
