# Supplementary material for: Investigating the Meat Pathway as a Source of Human Nontyphoidal Salmonella Bloodstream Infections and Diarrhea in East Africa
Source: Clin Infect Dis. 2020 Aug 10;73(7):e1570–8. doi: 10.1093/cid/ciaa1153 (PMC8492120; doi:10.1093/cid/ciaa1153)
Supplement: ciaa1153_suppl_Supplementary_Table_S3 [file ciaa1153_suppl_supplementary_table_s3.docx]

**Supplementary Table 3. *Salmonella* diversity indices by sample type, East Africa, 2007-17**

| **Source** | **Simpson (1-D)*** | | **Shannon (1-D)** | |
| --- | --- | --- | --- | --- |
|  | **Value** | **(95% CI)** | **Value** | **(95% CI)** |
| **Poultry farm environment** | **0.89** | **(0.85-0.93)** | **2.33** | **(2.10-2.56)** |
| **Poultry cloaca** | **0.86** | **(0.80-0.91)** | **2.06** | **(1.88-2.23)** |
| **Slaughter and butcher environment** | **0.93** | **(0.92-0.95)** | **2.97** | **(2.81-3.13)** |
| **Cattle intestinal** | **0.84** | **(0.71-0.96)** | **1.89** | **(1.52-2.26)** |
| **Goat intestinal** | **0.86** | **(0.78-0.94)** | **2.03** | **(1.73-2.32)** |
| **Cattle carcass** | **0.82** | **(0.71-0.93)** | **1.75** | **(1.41-2.09)** |
| **Goat carcass** | **0.70** | **(0.57-0.83)** | **1.28** | **(0.95-1.61)** |
| **Cattle meat** | **0.93** | **(0.91-0.95)** | **3.03** | **(2.84-3.23)** |
| **Goat meat** | **0.90** | **(0.86-0.94)** | **2.55** | **(2.31-2.80)** |
| **Human feces** | **0.88** | **(0.84-0.91)** | **2.52** | **(2.28-2.75)** |
| **Human blood** | **0.55** | **(0.49-0.60)** | **0.95** | **(0.76-1.14)** |
|  |  |  |  |  |

D = diversity

*For Simpson’s index of diversity (1-D), values approaching one represent high diversity and values approaching zero represent low diversity.
