# Supplementary material for: Investigating the Meat Pathway as a Source of Human Nontyphoidal Salmonella Bloodstream Infections and Diarrhea in East Africa
Source: Clin Infect Dis. 2020 Aug 10;73(7):e1570–8. doi: 10.1093/cid/ciaa1153 (PMC8492120; doi:10.1093/cid/ciaa1153)
Supplement: ciaa1153_suppl_Supplementary_Table_S4 [file ciaa1153_suppl_supplementary_table_s4.docx]

**Supplementary Table 4. Antimicrobial susceptibility testing by class, drug, and resistance genes of *Salmonella*, East Africa, 2007-17**

| **Antimicrobial class** | **Genes detected / phenotypic antimicrobials tested** | **Interpretation** | **Poultry farm environment** | | **Poultry cloaca** | | **Slaughter and butcher environment** | | **Cattle** | | **Goat** | | **Human faeces** | | **Human blood** | | **Total** | |
| --- | --- | --- | --- | --- | --- | --- | --- | --- | --- | --- | --- | --- | --- | --- | --- | --- | --- | --- |
|  |  |  | **n/n** | **(%)** | **n/n** | **(%)** | **n/n** | **(%)** | **n/n** | **(%)** | **n/n** | **(%)** | **n/n** | **(%)** | **n/n** | **(%)** | **n/n** | **(%)** |
| **Aminoglycoside** | *aac(3)-Id_1, aac(3)-IIa_1, aac(3)-IId_1, aac(6')Ib-cr_1, aadA1_1, aadA1_4, aadA1_5, aadA2_2, aadA7_1, strA_1, strA_2, strA_4, strB_1* |  | 11/34 | (32.4) | 11/39 | (28.2) | 2/97 | (2.1) | 6/124 | (4.8) | 4/73 | (5.5) | 28/90 | (31.1) | 71/82 | (86.6) | 133/539 | (24.7) |
|  | Gentamicin | S | 27/28 | (96.4) | 28/34 | (82.4) | 46/46 | (100) | 83/87 | (95.4) | 51/51 | (100) |  |  |  |  | 235/246 | (95.5) |
|  |  | I | 0/28 | (0.0) | 0/34 | (0.0) | 0/46 | (0.0) | 0/87 | (0.0) | 0/51 | (0.0) |  |  |  |  | 0/246 | (0) |
|  |  | R | 1/28 | (3.6) | 6/34 | (17.6) | 0/46 | (0.0) | 4/87 | (4.6) | 0/51 | (0.0) |  |  |  |  | 11/246 | (4.5) |
| **Beta-lactam** | *blaCTX-M-15_23, blaOXA-1_1, blaTEM-104_1, blaTEM-105_1, blaTEM-141_1, blaTEM-1B_1, blaTEM-1D_83* |  | 5/34 | (14.7) | 3/39 | (7.7) | 4/97 | (4.1) | 2/124 | (1.6) | 1/73 | (1.4) | 22/90 | (24.4) | 68/82 | (82.9) | 105/539 | (19.5) |
|  | Amoxicillin-clavulanate | S | 28/28 | (100) | 34/34 | (100) | 46/46 | (100) | 87/87 | (100) | 51/51 | (100) |  |  |  |  | 246/246 | (100) |
|  |  | I | 0/28 | (0.0) | 0/34 | (0.0) | 0/46 | (0.0) | 0/87 | (0.0) | 0/51 | (0.0) |  |  |  |  | 0/246 | (0) |
|  |  | R | 0/28 | (0.0) | 0/34 | (0.0) | 0/46 | (0.0) | 0/87 | (0.0) | 0/51 | (0.0) |  |  |  |  | 0/246 | (0) |
|  | Ampicillin | S | 28/28 | (100) | 34/34 | (100) | 45/46 | (97.8) | 85/87 | (97.7) | 51/51 | (100) |  |  |  |  | 241/246 | (98.0) |
|  |  | I | 0/28 | (0.0) | 0/34 | (0.0) | 0/46 | (0.0) | 0/87 | (0.0) | 0/51 | (0.0) |  |  |  |  | 0/246 | (0) |
|  |  | R | 0/28 | (0.0) | 0/34 | (0.0) | 1/46 | (2.2) | 2/87 | (2.3) | 0/51 | (0.0) |  |  |  |  | 5/246 | (2.0) |
|  | Ceftazidime | S | 28/28 | (100) | 34/34 | (100) | 46/46 | (100) | 87/87 | (100) | 51/51 | (100) |  |  |  |  | 246/246 | (100) |
|  |  | I | 0/28 | (0.0) | 0/34 | (0.0) | 0/46 | (0.0) | 0/87 | (0.0) | 0/51 | (0.0) |  |  |  |  | 0/246 | (0) |
|  |  | R | 0/28 | (0.0) | 0/34 | (0.0) | 0/46 | (0.0) | 0/87 | (0.0) | 0/51 | (0.0) |  |  |  |  | 0/246 | (0) |
|  | Ceftriaxone | S | 28/28 | (100) | 34/34 | (100) | 46/46 | (100) | 87/87 | (100) | 51/51 | (100) |  |  |  |  | 246/246 | (100) |
|  |  | I | 0/28 | (0.0) | 0/34 | (0.0) | 0/46 | (0.0) | 0/87 | (0.0) | 0/51 | (0.0) |  |  |  |  | 0/246 | (0) |
|  |  | R | 0/28 | (0.0) | 0/34 | (0.0) | 0/46 | (0.0) | 0/87 | (0.0) | 0/51 | (0.0) |  |  |  |  | 0/246 | (0) |
| **Fosfomycin** | *fosA_15* |  | 3/34 | (8.8) | 0/39 | (0.0) | 4/97 | (4.1) | 1/124 | (0.8) | 1/73 | (1.4) | 0/90 | (0.0) | 0/82 | (0.0) | 9/539 | (1.7) |
| **Macrolide** | *mef(B)_1* |  | 4/34 | (11.8) | 2/39 | (5.1) | 0/97 | (0.0) | 0/124 | (0.0) | 0/73 | (0.0) | 0/90 | (0.0) | 1/82 | (1.2) | 7/539 | (1.3) |
| **Phenicol** | *catA1_1, catA2_1, catB3_1, catB4_1, cmlA1_1* |  | 9/34 | (26.5) | 12/39 | (30.8) | 8/97 | (8.2) | 10/124 | (8.1) | 2/73 | (2.7) | 25/90 | (27.8) | 70/82 | (85.4) | 136/539 | (25.2) |
|  | Chloramphenicol | S | 24/28 | (85.7) | 33/34 | (97.1) | 46/46 | (100) | 85/87 | (97.7) | 51/51 | (100) |  |  |  |  | 239/246 | (97.2) |
|  |  | I | 0/28 | (0.0) | 0/34 | (0.0) | 0/46 | (0.0) | 2/87 | (2.3) | 0/51 | (0.0) |  |  |  |  | 2/246 | (0.8) |
|  |  | R | 4/28 | (14.3) | 1/34 | (2.9) | 0/46 | (0.0) | 0/87 | (0.0) | 0/51 | (0.0) |  |  |  |  | 5/246 | (2.0) |
| **Quinolone** | *oqxB_1, qnrB1_1, qnrS1_1* |  | 5/34 | (14.7) | 3/39 | (7.7) | 0/97 | (0.0) | 2/124 | (1.6) | 0/73 | (0.0) | 0/90 | (0.0) | 1/82 | (1.2) | 11/539 | (2.0) |
|  | *gyrA* D87N SNP |  | 6/34 | (17.6) | 7/39 | (17.9) | 0/97 | (0.0) | 2/124 | (1.6) | 0/73 | (0.0) | 0/90 | (0.0) | 3/82 | (3.7) | 18/539 | (3.3) |
|  | *gyrB* S464Y SNP |  | 0/34 | (0.0) | 0/39 | (0.0) | 0/97 | (0.0) | 0/124 | (0.0) | 0/73 | (0.0) | 1/90 | (1.1) | 1/82 | (1.2) | 2/539 | (0.4) |
|  | *parC* S80I SNP |  | 2/34 | (5.9) | 7/39 | (17.9) | 0/97 | (0.0) | 4/124 | (3.2) | 0/73 | (0.0) | 0/90 | (0.0) | 0/82 | (0.0) | 13/539 | (2.4) |
|  | Nalidixic acid | S | 20/28 | (71.4) | 22/34 | (64.7) | 46/46 | (100) | 81/87 | (93.1) | 50/51 | (98.0) |  |  |  |  | 219/246 | (89.0) |
|  |  | I | 2/28 | (7.1) | 0/34 | (0.0) | 0/46 | (0.0) | 0/87 | (0.0) | 0/51 | (0.0) |  |  |  |  | 2/246 | (0.8) |
|  |  | R | 6/28 | (21.4) | 12/34 | (35.3) | 0/46 | (0.0) | 6/87 | (6.9) | 1/51 | (2.0) |  |  |  |  | 25/246 | (10.2) |
|  | Ciprofloxacin | S | 18/28 | (64.3) | 21/34 | (61.8) | 46/46 | (100) | 66/87 | (75.9) | 32/51 | (62.7) |  |  |  |  | 183/246 | (74.4) |
|  |  | I | 8/28 | (28.6) | 6/34 | (17.6) | 0/46 | (0.0) | 17/87 | (19.5) | 19/51 | (37.3) |  |  |  |  | 50/246 | (20.3) |
|  |  | R | 2/28 | (7.1) | 7/34 | (20.6) | 0/46 | (0.0) | 4/87 | (4.6) | 0/51 | (0.0) |  |  |  |  | 13/246 | (5.3) |
| **Sulphonamide** | *sul1_2, sul2_1, sul2_10, sul2_13, sul2_14, sul2_19, sul2_2, sul2_3, sul2_6, sul3_2* |  | 9/34 | (26.5) | 11/39 | (28.2) | 4/97 | (4.1) | 8/124 | (6.5) | 3/73 | (4.1) | 27/90 | (30.0) | 69/82 | (84.1) | 131/539 | (24.3) |
| **Tetracycline** | *tet(A)_2, tet(A)_3, tet(A)_4, tet(B)_4, tet(C)_9* |  | 8/34 | (23.5) | 9/39 | (23.1) | 4/97 | (4.1) | 10/124 | (8.1) | 5/73 | (6.8) | 18/90 | (20.0) | 48/82 | (58.5) | 102/539 | (18.9) |
|  | Tetracycline | S | 22/28 | (78.6) | 25/34 | (73.5) | 45/46 | (97.8) | 81/87 | (93.1) | 47/51 | (92.2) |  |  |  |  | 220/246 | (89.4) |
|  |  | I | 0/28 | (0.0) | 0/34 | (0.0) | 0/46 | (0.0) | 0/87 | (0.0) | 0/51 | (0.0) |  |  |  |  | 0/246 | (0) |
|  |  | R | 6/28 | (21.4) | 9/34 | (26.5) | 1/46 | (2.2) | 6/87 | (6.9) | 4/51 | (7.8) |  |  |  |  | 26/246 | (10.6) |
| **Trimethoprim** | *dfrA1_30, dfrA12_1, dfrA14_1, dfrA7_1* |  | 2/34 | (5.9) | 2/39 | (5.1) | 0/97 | (0.0) | 1/124 | (0.8) | 2/73 | (2.7) | 21/90 | (23.3) | 69/82 | (84.1) | 97/539 | (18.0) |
|  | Trimethoprim-sulfamethoxazole | S | 26/28 | (92.9) | 32/34 | (94.1) | 46/46 | (100) | 87/87 | (100) | 49/51 | (96.1) |  |  |  |  | 240/246 | (97.6) |
|  |  | I | 0/28 | (0.0) | 0/34 | (0.0) | 0/46 | (0.0) | 0/87 | (0.0) | 0/51 | (0.0) |  |  |  |  | 0/246 | (0) |
|  |  | R | 2/28 | (7.1) | 2/34 | (5.9) | 0/46 | (0.0) | 0/87 | (0.0) | 2/51 | (3.9) |  |  |  |  | 6/246 | (2.4) |

S = susceptible; I = intermediate; R = resistant
